# Estrogen Receptor Blockade Potentiates Immunotherapy for Liver Metastases by Altering the Liver Immunosuppressive Microenvironment

**DOI:** 10.1158/2767-9764.CRC-24-0196

**Published:** 2024-08-08

**Authors:** Yasmine Benslimane, Kevin Amalfi, Sara Lapin, Stephanie Perrino, Pnina Brodt

**Affiliations:** 1 Division of Experimental Medicine, Department of Medicine, McGill University, Montreal, Canada.; 2 The Research Institute of the McGill University Health Center, Montreal, Canada.; 3 Department of Microbiology and Immunology, McGill University, Montreal, Canada.; 4 Department of Surgery, McGill University, Montreal, Canada.; 5 Department of Oncology, McGill University, Montreal, Canada.

## Abstract

Liver metastases (LM) remain a major cause of cancer-related death and are a major clinical challenge. LM and the female sex are predictors of a poorer response to immunotherapy but the underlying mechanisms remain unclear. We previously reported on a sexual dimorphism in the control of the tumor microenvironment (TME) of colorectal carcinoma liver metastases (CRCLM) and identified estrogen as a regulator of an immunosuppressive TME in the liver. Here we aimed to assess the effect of estrogen deprivation on the cytokine/chemokine profile associated with CRCLM, using a multiplex cytokine array and the RNAscope technology, and its effects on the innate and adaptive immune responses in the liver. We also evaluated the benefit of combining the selective estrogen-receptor degrader Fulvestrant with immune checkpoint blockade for the treatment of CRCLM. We show that estrogen depletion altered the cytokine/chemokine repertoire of the liver, decreased macrophage polarization, as reflected in reduced accumulation of tumor infiltrating M2 macrophages and increased the accumulation of CCL5^+^/CCR5^+^ CD8^+^ T and NKT cells in the liver TME. Similar results were obtained in a murine pancreatic ductal adenocarcinoma model. Importantly, treatment with Fulvestrant also increased the accumulation of CD8^+^CCL5^+^, CD8^+^CCR5^+^ T and NK cells in the liver TME and enhanced the therapeutic benefit of anti-PD1 immunotherapy, resulting in a significant reduction in the outgrowth of LM. Taken together, our results show that estrogen regulates immune cell recruitment to the liver and suggest that inhibition of estrogen action could potentiate the tumor-inhibitory effect of immunotherapy in hormone-independent and immunotherapy-resistant metastatic cancer.

**Significance:**

The immune microenvironment of the liver plays a major role in controlling the expansion of hepatic metastases and is regulated by estrogen. We show that treatment of tumor-bearing mice with an estrogen receptor degrader potentiated an anti-metastatic effect of immunotherapy. Our results provide mechanistic insight into clinical findings and a rationale for evaluating the efficacy of combination anti-estrogen and immunotherapy for prevention and/or treatment of hepatic metastases in female patients.

## Introduction

Metastasis is the primary cause of cancer-related mortality and remains a clinical challenge. The liver is a major site of metastases from cancers of the gastrointestinal (GI) tract such as colorectal carcinoma and also from non-GI cancers such as dermal and uveal melanoma and breast carcinoma. This is due, primarily, to the liver’s double blood supply from the hepatic artery and the portal vein, but also to the immuno-tolerant microenvironment (ME) of the liver. Up to 50% of patients with colorectal carcinoma develop liver metastases (CRCLM; ref. 1). In the United States, the incidence of colorectal carcinoma in adults 50 years and older decreased by 2% annually from 2014 to 2018, while increasing by 1.5% annually in adults younger than 50 years of age (2). Colorectal carcinoma has consequently become the fourth most diagnosed cancer among 30 to 39 years old men and women (2). Furthermore, male patients had a better 8-year overall survival (OS) than female patients with left or right colon cancer and with synchronous or metachronous LM (3). In patients diagnosed with advanced stage colorectal carcinoma, the 5-year survival rate currently stands at 12% (4). To date, the most effective treatment option is surgical resection of LM. However, only approximately 20% of patients with CRCLM are eligible for partial hepatectomy, generally due to size, number, and location of the metastases, and relapse can occur in up to 75% of resected patients (5, 6). To improve these statistics, combinatorial therapies that also target the pro-metastatic tumor microenvironment of the liver are actively being sought.

A sexual dimorphism in immunity has been well documented (7). However, the impact of sex on patients’ response to cancer immunotherapy (IT) is not yet fully understood. While immune checkpoint inhibitors (ICI) have extended patient survival across multiple cancer types, meta-analysis of 27 randomized clinical trials revealed that there was a superior overall survival in male than in female patients receiving ICI treatment, regardless of the protocol, the type of drug administered or the type of malignancy (8). Moreover, the presence of liver metastases, but not metastases in other organs, reduced IT efficacy in the clinical setting and this was also demonstrated in preclinical models (9). Thus, a better understanding of the role of sex and in particular, sex hormones, in regulating the response of liver metastatic disease to ICIs is essential in order to personalize disease management and improve the outcome for patients of both sexes.

Liver-resident macrophages (Kupffer cells) and recruited monocyte/macrophages can play opposing roles in the process of liver colonization by metastatic tumor cells and can either inhibit or promote metastatic expansion, depending on their phenotype (10). These properties are determined by a process of macrophage polarization on a spectrum from a pro-inflammatory and tumor-inhibitory (M1) phenotype to an anti-inflammatory pro-tumorigenic (M2) phenotype, a process that is regulated by cytokines, chemokines, and growth factors released by the tumor cells and other immune cells in the TME (11). These factors also regulate the recruitment and activity of other innate immune cells such as liver NK and NKT cells.

We recently documented a sexual dimorphism in the tumor immune microenvironment (TIME) of LM and identified estrogen as a central regulator of this tumor microenvironment (TME; ref. 12). More specifically, we identified an estrogen-regulated accumulation of myeloid-derived suppressor cells (MDSC) in the liver TME that impeded CD8^+^ T-cell–mediated anti-tumor immunity. Namely, we have shown that in mice depleted of estrogen by ovariectomy, the accumulation of MDSC, particularly granulocytic MDSC (G-MDSC) in the liver was markedly reduced, and this was associated with increased interferon gamma (IFNγ) and granzyme B production in recruited CD8^+^ T cells and reduced TNFR2, indolamine-2,3-dioxygenase (IDO), and tryptophan-2,3-dioxygenase (TDO) and Serpin B9 expression levels in the liver. Furthermore, in estrogen-depleted mice, experimental liver metastasis from colorectal carcinoma, pancreatic, and lung carcinoma was significantly reduced, and this reduction was also observed in mice injected with the selective estrogen receptor modulator (SERM), Tamoxifen (12).

In the present study, we analyzed the effect of estrogen depletion on the cytokine/chemokine profile of the liver, with a focus on factors that may regulate macrophage polarization and the recruitment and function of NK cells. Moreover, we evaluated the effect of combining an anti-PD-1 antibody with the selective estrogen receptor degrader (SERD) Fulvestrant (Fulv) on the TME and colon carcinoma liver metastasis. Our results show that estrogen depletion, or blockade of its activity, reprogrammed the chemokine/cytokine repertoire in the TME, increasing the proportion of pro-inflammatory macrophages and also enhancing the recruitment and cytotoxicity of a subset of NK cells in the liver TME.

## Material and Methods

### Animals

All mouse experiments were carried out in strict adherence to the guidelines of the Canadian Council on Animal Care “Guide to the Care and Use of Experimental Animals” and under the conditions and procedures approved by the Animal Care Committee of McGill University (AUP number: 5260). Mouse experiments were performed mainly in 6- to 12-week-old female C57BL/6 mice bred in the animal facility of the RI-MUHC (Glen Site) and all control mice (SHAM, and vehicle-treated) were age-matched to treated mice in all the experiments.

### Cells

The murine colorectal carcinoma MC-38 cells are syngeneic to the C57BL/6 strain. Their origins and metastatic properties have been described in detail previously (12). MC-38 cells were originally from an NCI repository and were obtained as a kind gift from Dr. Shoshana Yakar (New York University, NY). They were authenticated by Didion and colleagues using single-nucleotide polymorphism profiling (13). The murine pancreatic ductal adenocarcinoma (PDAC) line KPC FC1199, referred to here as FC1199, was generated in the Tuveson laboratory (Cold Spring Harbor Laboratory, New York, USA) from a PDAC tumor that arose in the genetically engineered Kras G12D/+; p53R172H/+; Pdx1 Cre (KPC) mouse of a pure C57BL/6 background, as described elsewhere (14) and were a kind gift from Dr. Andrew Lowy (Moores Cancer Center, La Jolla, CA). The cells were tested for common murine pathogens and for mycoplasma contamination (last mycoplasma test performed in August 2021 using a Mycoplasma PCR detection Kit – Abcam), as per the McGill University Animal Care Committee and the McGill University Biohazard Committee guidelines. The cells were maintained as a frozen stock and cultured *in vitro* for up to 4 weeks only, prior to use in the *in vivo* experiments, to minimize contamination, genetic drifts, and changes to their metastatic phenotypes. They were cultured in DMEM medium (Wisent), supplemented with 4 mmol/L L-glutamine, 4.5 g/L glucose, 100 U/mL penicillin, and 100 μg/mL streptomycin solution (Sigma-Aldrich, Burlington, Massachusetts, USA), also containing 2 g/L sodium pyruvate and 10% fetal bovine serum (FBS; Wisent) and incubated at 37°C in a humidified incubator with 5% CO_2_.

### Ovariectomy

Mouse ovariectomy was performed according to the McGill University Standard Operating Procedures (SOP No. 206.01) and with the approval of the Animal Care Committee of McGill University and the Research Institute of the McGill University Health Center. Briefly, female mice aged 5 to 7 weeks were administered carprofen (Rimadyl; 20 mg/kg; s.c.) and buprenorphine (Chiron Compounding Pharmacy Inc., 1 mg/kg; s.c. slow-release), 30 minutes prior to surgery and anesthetized using isoflurane. Animals were placed in sternal recumbence; their backs shaved and then disinfected using 70% ethanol and a 2% chlorhexidine solution. For each ovary, a single 0.5-cm dorsal flank incision was made, penetrating the abdominal cavity. The exposed ovaries were then removed by cauterization. The incisions in the peritoneal wall were sutured and incisions in the skin were closed with metal clips. Administration of carprofen (s.c.) and buprenorphine (s.c.) provided postoperative analgesia. In control, sham-operated mice, two 0.5-cm dorsal flank incisions penetrating the abdominal cavity were made, but the ovaries were not removed.

### Hormone replacement

β-estradiol (Sigma-Aldrich) was thoroughly mixed in sterile sesame oil (Sigma-Aldrich) at a concentration of 18 to 36 μg/mL. Placebo capsules were filled with sesame oil only. Capsules were prepared from silastic tubing and plugged with 3 mm wooden applicator sticks. The capsules were filled with the hormone solution, capped, and incubated overnight in the remaining hormone/oil solution to equilibrate, then implanted subcutaneously in female mice that were ovariectomized 14 days earlier. Reconstitution of serum estrogen levels in mice treated in this manner was previously confirmed (12).

### Immunostaining and confocal microscopy

C57BL/6 female mice were injected via the intrasplenic/portal route with 5 × 10^5^ MC-38 cells (or as indicated) and the livers perfused at the time intervals indicated, first with 3 mL PBS and then with 4 mL of a 4% paraformaldehyde solution. The perfused livers were placed in a 4% paraformaldehyde solution in PBS for 48 hours and then in 30% sucrose for an additional 48 hours, before they were stored at −80°C until used. For immunostaining, 15 μm cryostat sections were prepared, incubated first in a blocking solution (1% bovine serum albumin and 1% FBS in PBS), and then for 1 hour each with the primary antibodies (for a list of antibodies, see Supplementary Table S1), and the appropriate Alexa Fluor–conjugated secondary antibodies, all at room temperature. The sections were mounted in the Prolong Gold antifade reagent (Molecular Probes, Eugene, Oregon, USA) and confocal images were captured with a Zeiss LSM-780 microscope with a spectrum detection capability. Immunostained cells were quantified blindly in at least 10 images acquired per section, per experimental group.

### Hepatic lymphocytes isolation protocol

To analyze early changes in the TIME, mice were injected with 5 × 10^5^ tumor cells via the intrasplenic/portal route and the livers removed 6 to 9 days later (as indicated). Liver homogenates were prepared in cold PBS and filtered through a stainless-steel mesh using a plunger. The filtrates were centrifuged at 60 *g* to separate the hepatocytes, the supernatants containing the non-parenchymal cell fraction centrifuged at 480 *g*, the pellets resuspended in 10 mL of a 37.5% Percoll solution in HBSS containing 100 U/mL heparin and centrifuged at 850 *g* for 30 minutes to obtain the immune cell–rich fraction. Prior to flow cytometry (FC), red blood cells were removed using the ACK (ammonium–chloride–potassium) solution and 10^6^ cells were immunostained with the indicated antibodies (see antibodies listed in Supplementary Table S2). Single cells were gated based on size (forward scatter), granularity (side scatter), and viability using an eFluor 450 fixable dye (eBioscience, ThermoFisher). Data acquisition was performed with a BD FACS Diva software and the data analyzed using the FlowJo software.

### Hepatic macrophages isolation protocol

To analyze macrophages polarization in the TME, mice were injected with 5 × 10^5^ MC-38 cells via the intrasplenic/portal route, and the livers removed 8 to 12 days later (as indicated). Livers were minced and enzymatically digested using 0.1% Collagenase IV (from *Clostridium histolyticum*, Sigma-Aldrich) in cRPMI for 30 minutes at 37°C with mixing. The liver homogenates were filtered through a 74 μm mesh filter to remove debris and undigested tissue, the filtrate centrifuged for 5 minutes at 300 *g* in a 4°C centrifuge and the cells washed twice in cRPMI. Hepatocytes were removed by a 3 minutes centrifugation at 60 *g* and the supernatants containing the macrophages centrifuged at 300 *g* for 5 minutes at 4°C. Prior to FC, red blood cells were removed using the ACK (ammonium–chloride–potassium) solution and 1 × 10^6^ cells were immunostained with the indicated antibodies (listed in Supplementary Table S2). Single cells were gated based on size (forward scatter), granularity (side scatter), and viability using an eFluor 450 fixable dye (eBioscience, ThermoFisher). Data acquisition was performed with a BD FACS Diva software and the data analyzed using the FlowJo software.

### NK and NKT cells cytotoxicity assay

To analyze hepatic NKT and NK cell cytotoxicity, mice were injected with 5 × 10^5^ MC-38 tumor cells via the intrasplenic/portal route, and the livers removed 7 days later. One day prior to immune cells isolation, MC-38 cells were seeded and incubated with the Incucyte Cytotox Green Dye. (As per manufacturer’s guidelines, addition of the Incucyte Cytotox Dyes to normal healthy cells is non-perturbing to cell growth or morphology and will not yield a fluorescence increase, as entry and DNA-binding of Incucyte Cytotox Dye occurs in damaged cells only). The day after MC-38 seeding, liver homogenates were prepared as per the hepatic lymphocyte isolation protocol (described above). NK and NKT cells from OVX, SHAM, and OVX + E2 mice were isolated and FACS sorted based on the expression of CD3 and/or NK1.1 cell surface markers and co-incubated with MC-38-target cells in triplicates at a ratio 5:1 (NK or NKT: MC-38 cells). The cell mixture was incubated and analyzed continuously for 30 hours using the Incucyte system, and green fluorescent/dead MC-38 cells were recorded and normalized to time 0 hour.

### Multiplex cytokine array

A cytokine array assay was performed on livers derived from mice injected with 5 × 10^5^ MC-38 cells via the intrasplenic/portal route and sacrificed on day 12 post injection. Liver tissue was lyophilized and lysed using the RIPA buffer (50 mmol/L Tris-HCl, pH 8, 150 mmol/L NaCl, 0.1%Triton X-100, 0.1% SDS, and 0.5% sodium deoxycholate) supplemented with the Protease Inhibitor Cocktail (Roche cOmplete Mini, Sigma Aldrich Canada, Oakville, Ontario) for 30 minutes at 4°C. Total protein lysates were clarified by centrifugation at 13,000 *g* for 20 minutes. Protein was collected and expression profiles of cytokines and chemokines were analyzed with the Proteome Profiler Mouse Cytokine Array Panel A (R&D systems, Minneapolis, MN, USA), as per the manufacturer’s instructions. Pixel densities from SHAM-control mice were used to normalize the data.

### RNA extraction and qPCR

Total cellular RNA was extracted from snap-frozen liver fragments using the TRIzol reagent (Life Technologies, Inc., Burlington, Ontario, Canada), according to the manufacturer’s instructions. Two micrograms of RNA were reverse transcribed and the cDNA used for qPCR analysis with the primer sets listed in Supplementary Table S3.

### *In situ* hybridization by RNAscope

Freshly cut frozen sections were pretreated using the following specific optimal conditions: 1 hour fixation in 4% paraformaldehyde in PBS, 5 minutes target retrieval in citrate buffer, and 30 minutes incubation in protease III, as instructed by the manufacturer [Advanced Cell Diagnostics, Inc. (ACD) Newark, CA]. Following the standard ACD protocol (RNAscope Multiplex Fluorescent Reagent Kit v2, User Manual UM 323100), RNA-specific probes designed for different fluorescent detection channels to target multiple RNAs simultaneously were hybridized to the tissue sections. After a series of highly effective and specific signal amplifications, single-RNA transcripts for target genes appear as punctate dots in different fluorescent channels. Multiple images from each section were acquired using the Zeiss LSM780 confocal microscope and quantification was performed blindly using five images per condition.

### Experimental liver metastasis

Experimental LM were generated by intrasplenic/portal injection of 10^5^ tumor cells, followed by splenectomy, as we previously described (12). Animals were euthanized 18 to 21 days later, and visible metastases on the surfaces of the livers were enumerated and sized without prior fixation. Where indicated, fragments of the livers were also fixed in 10% phosphate-buffered formalin, paraffin embedded, and 7 μm sections hematoxylin and eosin (H&E) stained.

### Fulv treatment

Experimental liver metastases were generated by intrasplenic/portal injections of 10^5^ tumor cells, followed by splenectomy. Mice were inoculated subcutaneously three times weekly with the indicated concentration of Fulv (an AstraZeneca product, sold by Sigma-Aldrich) solubilized in DMSO and mixed in sterile sunflower seed oil (Sigma-Aldrich) or with sunflower seed oil + DMSO at the appropriate concentration that was used as a vehicle control. Mice were sacrificed on day 21 post-MC-38 injection for metastases enumeration.

### Combinatorial Fulv/anti-PD-1 treatment

Experimental liver metastases were generated as described above (day 0). On day 1, 20 mg/kg Fulv (Sigma) reconstituted in DMSO and injected in sterile sunflower seed oil or sunflower seed oil/DMSO were administered subcutaneously. The following day, 10 mg/kg of the anti-PD-1 antibody (clone RMP1-14, BioXcell, Lebanon, New Hampshire, USA) or 10 mg/kg of the IgG isotype control (BioXcell) were administered intraperitoneally. This was repeated on days 4 and 5, respectively, and thereafter weekly until day 15. The mice were sacrificed on day 18 post MC-38 injection, livers excised, and metastases enumerated as described above.

### Statistical analyses

The nonparametric Mann–Whitney test was used to analyze all metastasis data and a two-tailed Student *t* test was used to analyze *ex vivo* and *in vitro* data and the immunofluorescence results.

## Data Availability

The authors declare that the data supporting the findings of this study are available within the paper and its supplementary information files. Unprocessed (raw) data can be made available by the corresponding author upon reasonable request.

## Results

### Estrogen depletion enhances NKT cell recruitment and cytotoxic activity, and this can be reversed by estrogen supplementation

**Figure 1:**
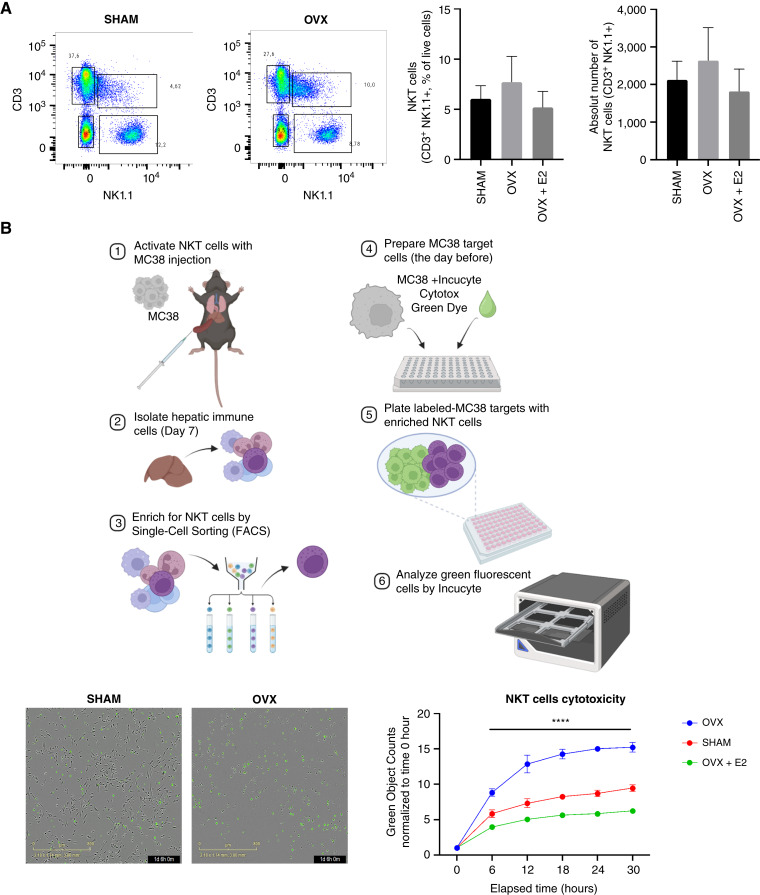
Estrogen depletion increases NKT cytotoxicity in the liver TME. FC was performed on hepatic immune cells isolated following injection of 5 × 10^5^ MC-38 cells and immunolabeled with the indicated antibodies. Shown in **A** are flow cytometric contour plots and the respective bar graphs (*n* = 4). Shown in **B** (top) is the workflow of the NKT cytotoxicity assay, in **B** (bottom-left) representative images of green fluorescent (dead) MC-38 cells after co-culture with NKT cells for 30 hours and in **B** (bottom right) quantification of dead MC-38 cells as measured over time, normalized to time 0 hour (*n* = 2–3). ****, *P* ≤ 0.0001; Two-way ANOVA. (Created with BioRender.com.)

The liver is host to the largest population of natural killer T (NKT) cells in the body (15). Liver NKT cells patrol the liver sinusoids to provide intravascular immune surveillance against foreign “invaders,” such as cancer cells (16, 17). They can mount a strong antitumor response and have become a major focus in the effort to develop effective cancer immunotherapy (18). Having previously observed a significant reduction in the number of liver metastases in estrogen depleted (ovariectomized, OVX) mice, regardless of histological tumor cell type (12), it was of interest to determine whether estrogen depletion altered the accumulation and function of these cytotoxic cells in the liver TME. To this end, we isolated hepatic immune cells (HIC) from OVX and sham-operated (SHAM control) mice for analysis by FC, 7 days post intrasplenic/portal inoculation of the murine colon cancer MC-38 cells. As an additional control, we used a third group, consisting of OVX mice in which estrogen levels were reconstituted by subcutaneous implantation of silastic capsules containing 18 to 36 μg/mL β-estradiol (OVX + E2), as we previously described (12). In OVX mice, we observed a minor increase in the accumulation of CD3^+^NK1.1^+^ NKT cells relative to SHAM controls and OVX + E2 mice (Fig. 1A). To determine whether estrogen withdrawal affected the cytotoxic potential of these cells, we used an optimized Incucyte-based cytotoxicity assay that enables visualization and quantification of cell death in real time. MC-38 cells labeled with the Incucyte Cytotox Green Dye were co-incubated with NKT cells that were isolated and FACS-sorted 6 days post tumor cell injection (Fig. 1B). We observed a significantly higher proportion of dead (green fluorescent) MC-38 cells following co-incubation with OVX, as compared to SHAM-derived NKT cells that were used at the same NKT:tumor cell ratio (Fig. 1B) and β-estradiol supplementation restored NKT cytotoxicity levels to those observed in SHAM controls (Fig. 1B), suggesting that estrogen reduced the cytotoxic potential of liver NKT cells. This effect was specific to the NKT subpopulation, as no significant difference was observed in the cytotoxic activity of CD3^−^NK1.1^+^ NK cells derived from OVX mice when compared to the controls (Supplementary Fig. S1).

### Estrogen depletion reprograms the cytokines/chemokines repertoire of the liver TME and enhances the expression of CCL5 and its cognate receptor CCR5

**Figure 2:**
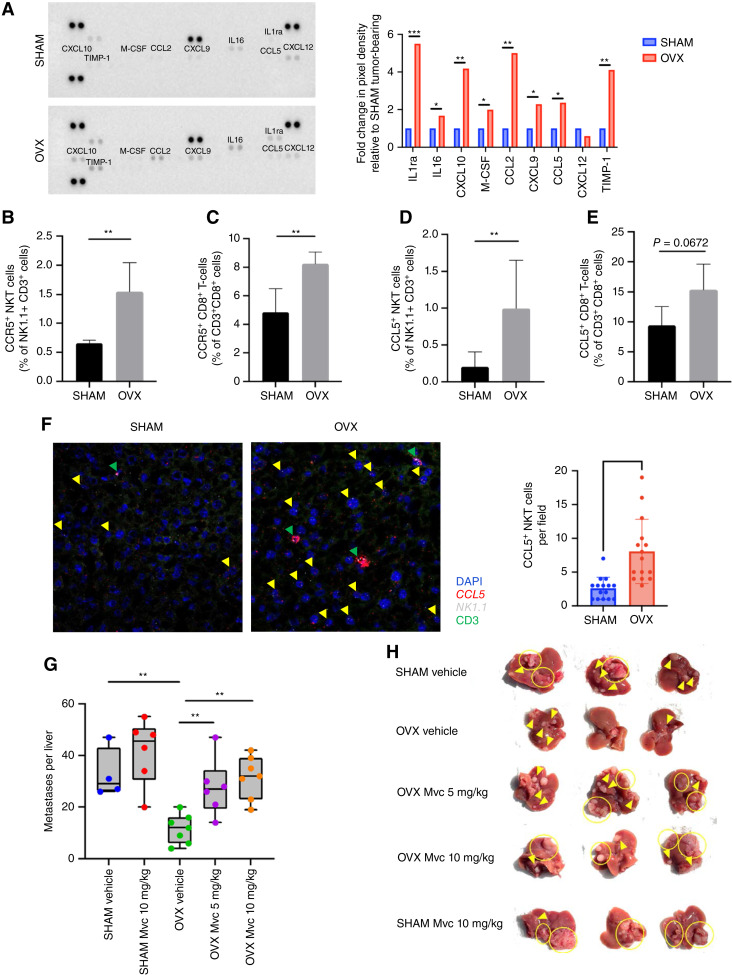
Estrogen depletion alters the cytokines/chemokines repertoire of the liver and increases expression of CCR5 and CCL5 in NKT and CD8^+^ T cells. **A,** Cytokine array analysis was conducted on whole liver protein lysates (four livers/group) derived from MC-38 tumor-bearing mice. Data were normalized to SHAM-operated values. In **B–E** are FC results for the indicated cell populations (*n* = 4). In **F** (left)—representative RNAscope images of frozen liver sections probed with CCL5 and NK1.1, followed by CD3 staining, and in **F** (right) image analysis results based on 15 fields per group (*n* = 3). Yellow arrows denote NK1.1^+^CCL5^+^CD3^+^ cells, and green arrows denote NK1.1^+^CD3^+^ cells with high CCL5 density. Experimental LM were generated by intrasplenic/portal injection of 10^5^ MC-38 cells. Intraperitoneal Mvc or vehicle injections began the following day and continued for 6 days. Mice were sacrificed and LM enumerated on day 18 post-inoculation. Results shown are of a representative experiment of two performed. The number of metastases per liver are shown in **G** (*n* = 4–7; bars indicate medians) and representative livers are shown in **H**. *, *P* ≤ 0.05; **, *P* ≤ 0.01; ***, *P* ≤ 0.001, Student *t* test (FC) and Mann–Whitney test (LM).

Chemokines and cytokines can affect the fate of metastatic cells either directly or indirectly, by regulating the type and activities of immune cells recruited into the TME (19). Having observed differences between the TIME of LM growing in estrogen-competent and depleted mice, it was of interest to identify the molecular mediators underpinning these shifts in immune cell accumulation in response to metastatic colorectal carcinoma cells. We therefore analyzed the cytokine/chemokine repertoires in livers obtained from these mice, following MC-38 inoculation, using a multiplex cytokine array. Intriguingly, the analysis revealed a general trend toward increased chemokine production in OVX, as compared to control mice for the majority of chemokines analyzed including CXCL10/IP-10 (four-fold), M-CSF/CSF-1 (two-fold), CCL2/MCP-1 (five-fold), CXCL9/MIG (two-fold), and CCL5/RANTES (two-fold; Fig. 2A). The cytokine array results were validated using qPCR confirming an increased expression of these chemokines in the livers of estrogen depleted mice (Supplementary Fig. S2A).

The chemokine CCL5 has been linked to NKT recruitment and activation and associated with the M1 macrophage phenotype (20, 21). We therefore analyzed further its role in the altered liver TME in estrogen-depleted mice. To determine the cellular source(s) of CCL5 and identify CCR5 expressing cells, we analyzed by FC HIC isolated 7 days post intrasplenic/portal MC-38 inoculation (for gating strategy see Supplementary Fig. S2B). We found increased CCR5 levels on CD3^+^NK1.1^+^ NKT and CD3^+^CD8^+^ T cells derived from OVX mice as compared to controls (Fig. 2B and C). We also found increased CCL5 levels in NKT cells (Fig. 2D) and to a lesser extent in CD8^+^ T cells (Fig. 2E) derived from OVX mice, consistent with the increased activity of NKT cells in estrogen-depleted mice. *In situ* hybridization using RNAscope confirmed high *ccl5* mRNA expression levels in liver *NK1.1*^*+*^CD3^*+*^ cells in OVX as compared to SHAM control mice (Fig. 2F; Supplementary Fig. S3). Similar trends were also observed in mice inoculated with the murine PDAC cells FC1199. We found increased accumulation of CCL5^+^ CD8^+^ T cells and to a lesser extent CCL5^+^ NKT cells in OVX mice injected with these cells and estrogen supplementation in OVX mice reversed these trends (Supplementary Fig. S4), suggesting that the changes observed in the IME were not tumor-type specific.

Maraviroc (Mvc) is a small-molecule CCR5 antagonist that is in clinical use for the treatment of CCR5-tropic HIV-1 infection (22). To ascertain the role of CCL5/CCR5 signaling in reducing the growth of metastases in OVX mice, we treated the mice with Mvc (5 or 10 mg/kg) or vehicle, daily for 6 days from day 2 onward post injection of 10^5^ MC-38 cells via the intrasplenic/portal route. We found that Mvc treatment significantly increased the number of CRCLM in OVX mice (Fig. 2G and H) and a more modest increase was also seen in control mice treated with Mvc, suggesting that CCL5 was contributing to the reduction in the growth of metastases in OVX mice.

### Increased expression of CXCR3 and its ligand CXCL10 in OVX mice also contributes to reprograming of the TIME

**Figure 3:**
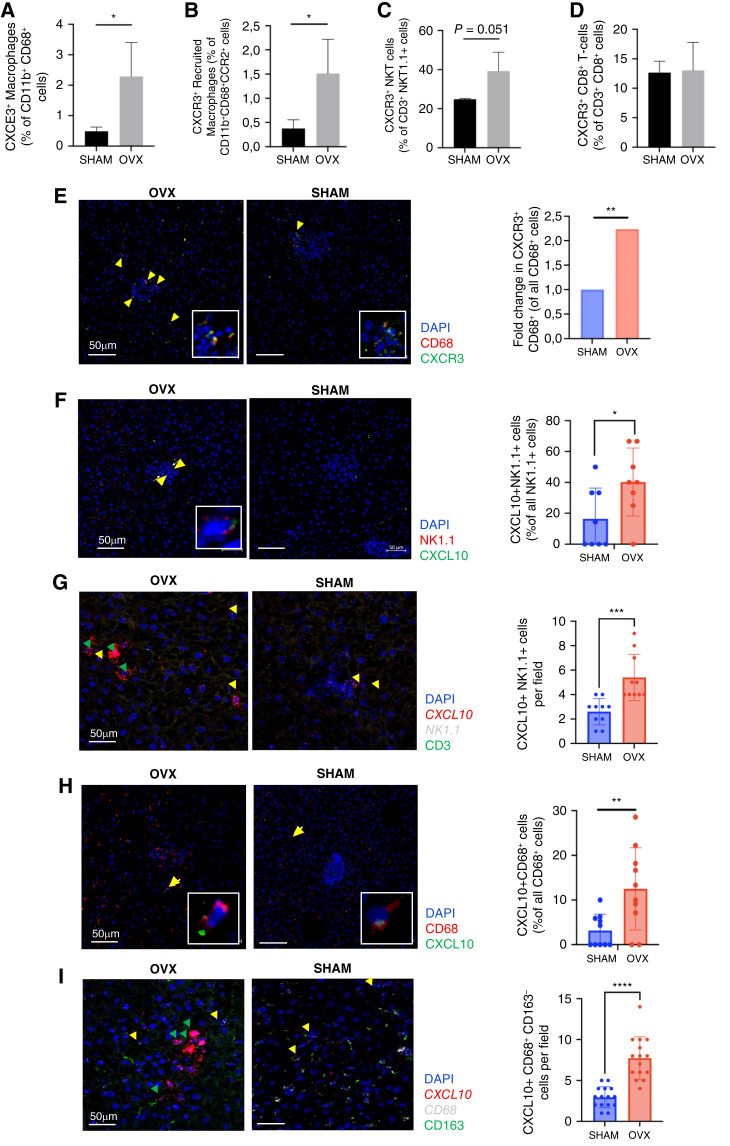
Increased CXCR3 expression in hepatic macrophages associated with early stages of the metastatic process. FC was conducted on hepatic immune cells from mice injected with 5 × 10^5^ MC-38 cells 8 days earlier. Shown in **A–D** are FC results for the indicated cell populations (*n* = 3–4). Confocal images (**E**, **F**, **H**) were acquired for 10 μm liver cryostat sections from these mice that were immunostained with the indicated antibodies and counterstained with 4′,6-diamidino-2-phenylindole. Cell counts per field (means ± SD, *n* = 10) are shown on the right. Representative RNAscope images were obtained from cryostat sections probed for CXCL10 expression in NKT (**G**) or macrophages (**I**), as indicated. Image analysis (**G**, **I**, right) was based on 15 fields per group (*n* = 2–3). Yellow arrows denote NK1.1^+^ or CD68^+^ cells expressing CXCL10, and green arrows denote cells with high CXCL10 expression. *, *P* ≤ 0.05; **, *P* ≤ 0.01; ***, *P* ≤ 0.001; ****, *P* ≤ 0.0001, Student *t* test.

The IFNγ-inducible chemokine CXCL10 has been implicated in T- and NKT-cell recruitment via receptor CXCR3 in autoimmune disease (23), various inflammatory conditions and cancer (24–26). Our cytokine array data revealed an upregulation of CXCR3 ligands CXCL9 and CXCL10 in OVX mice relative to controls (Fig. 2A). It was therefore of interest to investigate the role of this receptor/ligand interaction in NKT and T-cell recruitment and in liver metastasis in our model. FC analysis performed on HIC derived from MC-38 injected mice revealed in OVX mice an increase in CXCR3 levels, on CD68^+^ hepatic macrophages (Fig. 3A) and in particular, in the CD11b^+^CCR2^+^CD68^+^ recruited monocyte/macrophage subset (Fig. 3B), suggesting that this receptor may play a role in monocyte/macrophage recruitment to the liver in response to metastatic cancer cells. NKT, but not CD8^+^ T cells derived from OVX mice also expressed higher CXCR3 levels compared to controls (Fig. 3C and D), suggesting that this receptor may play a role in their recruitment to the TME in these mice.

Immunohistochemistry confirmed expression of CXCR3 on CD68^+^ macrophages (Fig. 3E). It also identified NK cells (Fig. 3F) and macrophages (Fig. 3H) as producers of CXCL10 in the liver IME and confirmed the increased production of this chemokine in these cells in OVX mice. This was further confirmed by *in situ* hybridization using RNAscope, revealing increased *CXCL10* mRNA expression in NK cells (Fig. 3G) and CD68^*+*^ macrophages in OVX mice (Fig. 3I), and a near absence of *CXCL10* expression in CD68^*+*^CD163^+^ cells (Fig. 3I), indicating a lack of CXCL10 expression in M2-like macrophages.

### Estrogen depletion alters the M1/M2 macrophage ratio in the liver TME

**Figure 4:**
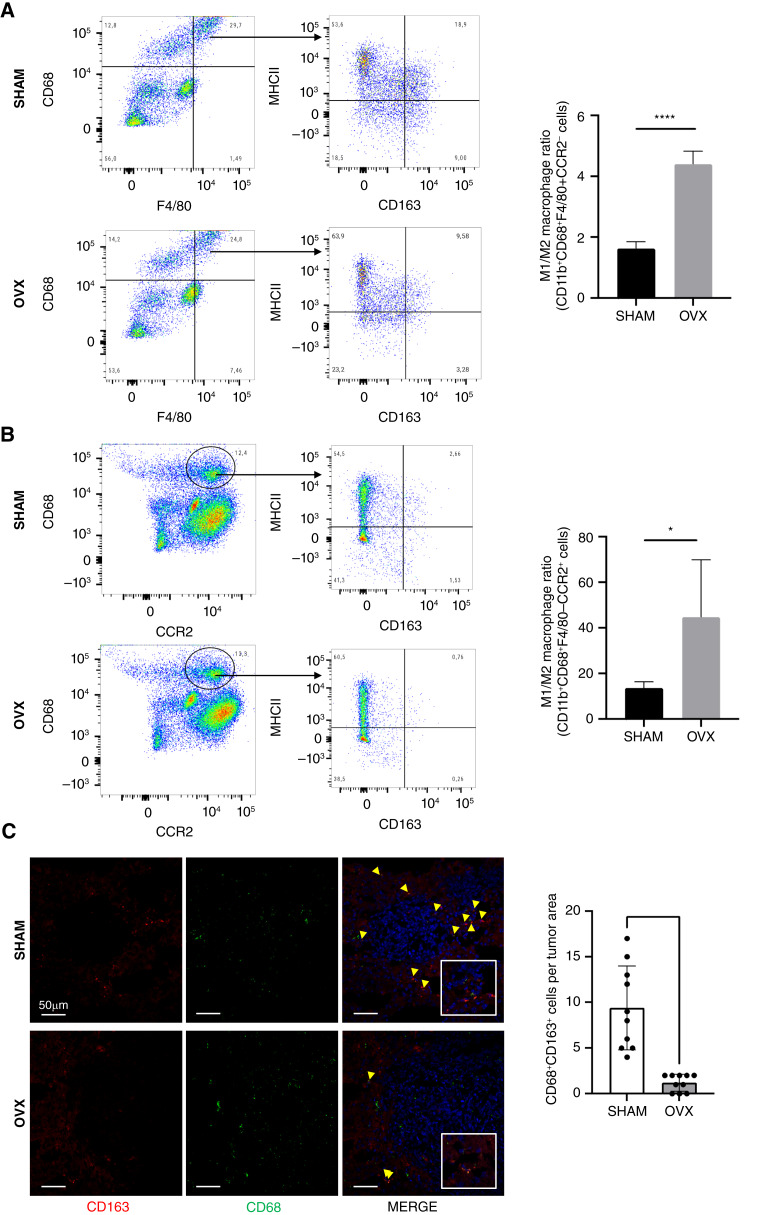
Estrogen depletion increases the M1/M2 macrophage ratio in both the recruited and liver-resident macrophage populations. FC was performed on macrophages isolated 12 days post injection of 5 × 10^5^ MC-38 cells and stained with the indicated antibodies. Shown in **A** and **B** are flow cytometric contour plots (left) and M1/M2 macrophage ratios (right, *n* = 4). Representative confocal images obtained from 15 μm liver sections labeled as indicated are shown in **C** (left) and cell counts per field (means ± SD, *n* = 10) are shown in **C** (right). *, *P* ≤ 0.05; ****, *P* ≤ 0.0001, Student *t* test.

The hepatic macrophage population consists of the liver-resident macrophages (Kupffer cells), and monocytes/macrophages recruited from the bone marrow; a process that occurs under normal physiological condition but is accelerated in response to pathological conditions (27). Tumor-associated macrophages (TAM) constitute a major component of the TME and can play opposing roles in the process of liver metastasis (28). Our finding that CXCR3 expression in OVX mice was significantly increased on a CD11b^+^CCR2^+^CD68^+^ macrophage subset and that CD163^−^ macrophages were a major source of CXCL10 in these mice was consistent with a potential shift in the polarization state of the TAM in the liver TME of these mice. To analyze the effect of estrogen depletion on the phenotype of hepatic macrophages in the TME, we analyzed by FC macrophages isolated from OVX and control mice, 12 days post MC-38 inoculation, using a combination of the cell surface markers CD11b, CD68, and F4/80 to identify Kupffer cells and Ly6C and CCR2 to identify recruited monocytes/macrophages. When MHCII and CD163 were added to identify M1-like and M2-like macrophages, respectively (29), and the ratio of CD11b^+^CD68^+^MHCII^+^ (M1): CD11b^+^CD68^+^CD163^+^ (M2) macrophages was calculated, we found that estrogen depletion altered the macrophage population in the TME, significantly increasing the M1/M2 macrophage ratio, in OVX, as compared to SHAM-control mice (Fig. 4A−C). This increased ratio was observed in both the resident and recruited macrophage populations (Fig. 4A and B respectively) and was reversed upon β-estradiol (E2) supplementation (Supplementary Fig. S5A and S5B), confirming the role of estrogen in the altered polarization state of the hepatic macrophages. Similar data were obtained when the M1-like macrophage markers, CD38 and TNF-α were used (Supplementary Fig. S5A and S5B, respectively). These results were confirmed by immunofluorescence staining of liver cryostat sections derived from some of these mice, where greater numbers of CD68^+^CD163^+^ M2-like cells could be observed in the periphery and infiltrating CRCLM in SHAM controls as compared to OVX mice (Fig. 4C). A similar trend in M1/M2 macrophage ratios was also seen in OVX mice inoculated with the PDAC FC1199 cells (Supplementary Fig. S5C), although the differences observed between groups were more minor. This suggests that the effect of estrogen withdrawal on macrophage polarization was not cancer-type specific, although its magnitude may vary.

### Fulv reprograms the immune microenvironment of the liver and inhibits CRCLM outgrowth

**Figure 5:**
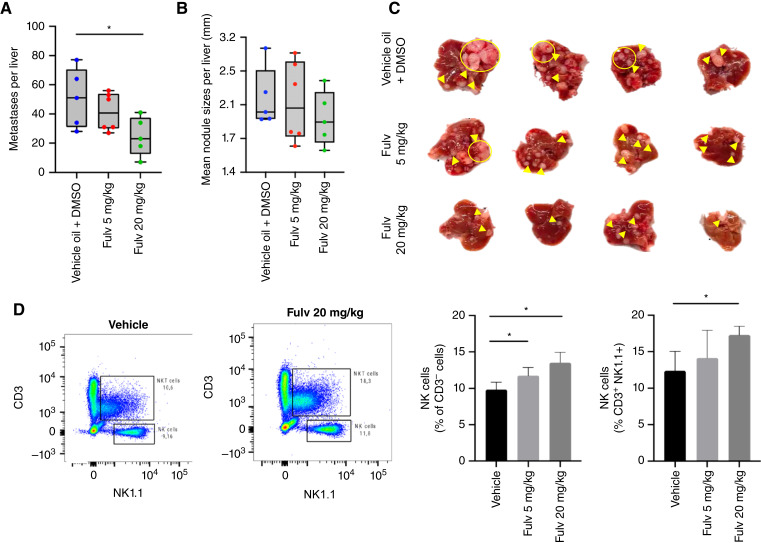
Fulv reprograms the liver TME and inhibits CRCLM outgrowth. Experimental LM were generated by intrasplenic/portal injection of 10^5^ MC-38 cells. Mice received eight subcutaneous injections of Fulv or vehicle on alternate days and were sacrificed 21 days post tumor inoculation. Results shown are of a representative experiment of two performed. In **A** are metastases counted per liver (*n* = 5–6), in **B** mean nodule size (mm; *n* = 5–6), and in **C** representative liver images. Hepatic immune cells were isolated in a separate experiment 7 days post-injection, stained with the indicated antibodies, and analyzed by FC. Shown in **D** (left) are representative FC contour plots (*n* = 3–4) and in **D** (right) cell counts (means ± SD). *, *P* ≤ 0.05, Mann–Whitney test (metastases) or Student *t* test (FC).

We have previously shown that estrogen depletion by ovariectomy, as well as Tamoxifen treatment markedly reduced the number and size of MC-38 liver metastases (12). Tamoxifen is a SERM with potential agonistic activity. To rule out any potential contribution of the agonist activity to outgrowth of metastases, we tested the effect on metastasis of the SERD Fulv that inhibits ER signaling by forming unstable SERD–ER complexes and targeting them for proteasomal degradation (30). This FDA approved drug is currently in clinical use for the management of hormone-receptor-positive advanced breast cancer (31). Female mice injected with MC-38 cells were administered Fulv (5 or 20 mg/kg) or vehicle (sunflower seed oil + DMSO) subcutaneously, three times weekly and liver metastases enumerated 21 days post tumor inoculation. In Fulv-treated mice, we observed a significant and dose-dependent reduction in the number of CRCLM, as compared to controls (Fig. 5A–C). Furthermore, when the TIME in the treated mice was analyzed, we found a dose-dependent increase in NKT cell frequencies in the Fulv-treated mice relative to controls (Fig. 5D), essentially mimicking the effect of surgical depletion of estrogen seen in OVX mice. In addition, in these mice, there was also a moderate increase in NK cell frequencies, particularly following treatment with the higher Fulv dose of 20 mg/kg.

### Fulvenhances the therapeutic effect of anti-PD1 immunotherapy against CRCLM

**Figure 6:**
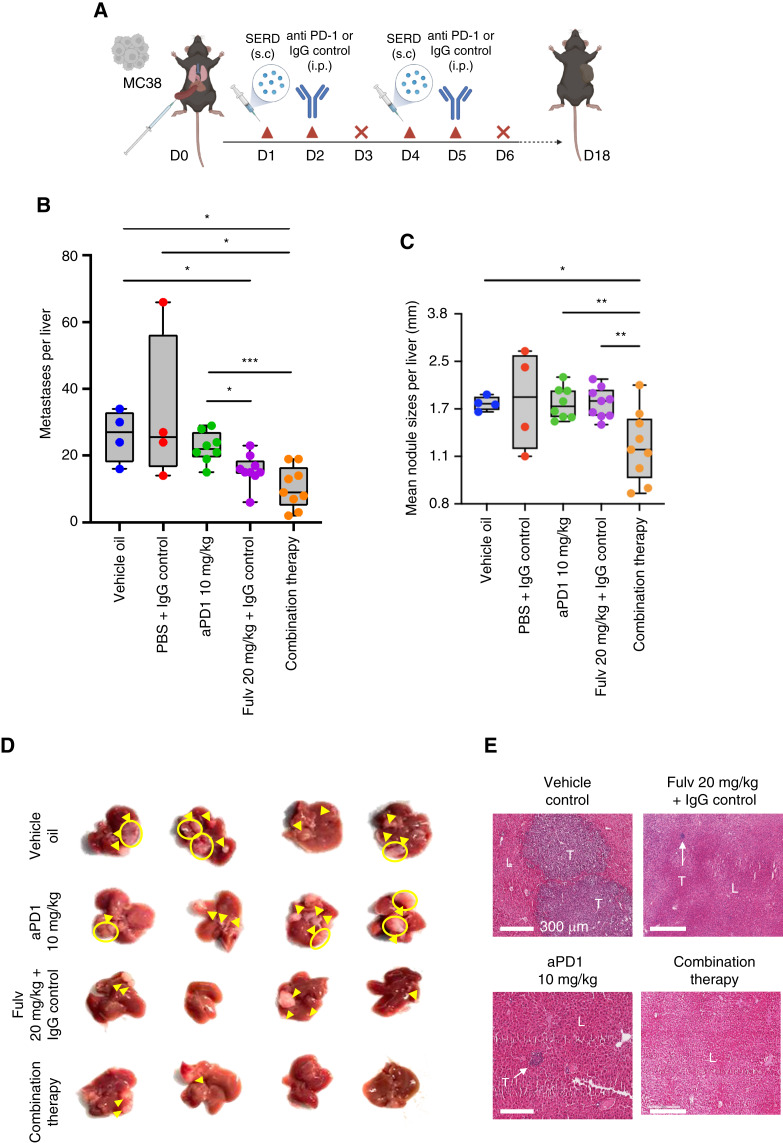
Fulv enhances the inhibitory effect of anti-PD-1 immunotherapy against CRCLM. Experimental LM were generated by intrasplenic/portal injection of 10^5^ MC-38 cells. Treatment consisted of subcutaneous injection of 20 mg/kg Fulv or vehicle on day 1 post-injection, followed by intraperitoneal administration of 10 mg/kg anti-PD-1 or control IgG on day 2, with no treatment on day 3, a protocol that was repeated until day 15 [as shown in **A**]. Mice were sacrificed and metastases enumerated on day 18. Shown in **B** are metastases counts per liver (*n* = 4–9, pooled data of two separate experiments), in **C** nodule size range (*n* = 4–9), in **D** representative liver images, and in **E** representative H&E-stained formalin-fixed, paraffin-embedded sections. *, *P* ≤ 0.05; **, *P* ≤ 0.01; ***, *P* ≤ 0.001, Mann–Whitney test.

Recent evidence suggests that the low efficacy of immunotherapy in the treatment of nonresponsive cancers can be enhanced by combining immune checkpoint inhibitors with other drugs that target immunosuppressive elements within the TME (32). The female sex has been identified as one of five variables that have significant negative associations with response to anti-PD-1 therapy (33). Our work and others have identified estrogen as a promoter of an immunosuppressive TME (34). Having identified Fulv as an effective modulator of the TME in the liver with consequences for metastatic expansion, we investigated whether a combinatorial therapy with Fulv and anti-PD-1 antibodies could enhance the therapeutic effect of the latter on MC-38 liver metastases. Female mice were injected with 10^5^ MC-38 cells via the intrasplenic/portal route and randomized to the following treatment groups: (i) Fulv vehicle (sunflower seed oil + DMSO); (ii) IgG isotype control; (iii) anti-PD-1 antibody 10 mg/kg; (iv) Fulv 20 mg/kg + IgG isotype control; and (v) 20 mg/kg Fulv + 10 mg/kg anti-PD-1 antibody (combination therapy) administered on alternate days (see diagram in Fig. 6A). The treatment continued for a total of five Fulv and anti-PD-1 injections each, and liver metastases were enumerated 18 days post tumor cell inoculation. We found that treatment with anti-PD-1 antibodies had no significant effect on the number or size of liver metastases while, as expected, Fulv reduced the number of LM, in comparison to both control groups. Notably, a further reduction in both the number and size of LM was observed when Fulv was combined with anti-PD-1 antibody treatment (Fig. 6B–E), indicating that Fulv could potentiate the inhibitory effect of IT on metastatic outgrowth in this model.

### Combinatorial Fulv and anti-PD-1 antibody treatment potentiates an antitumor response in the liver

**Figure 7:**
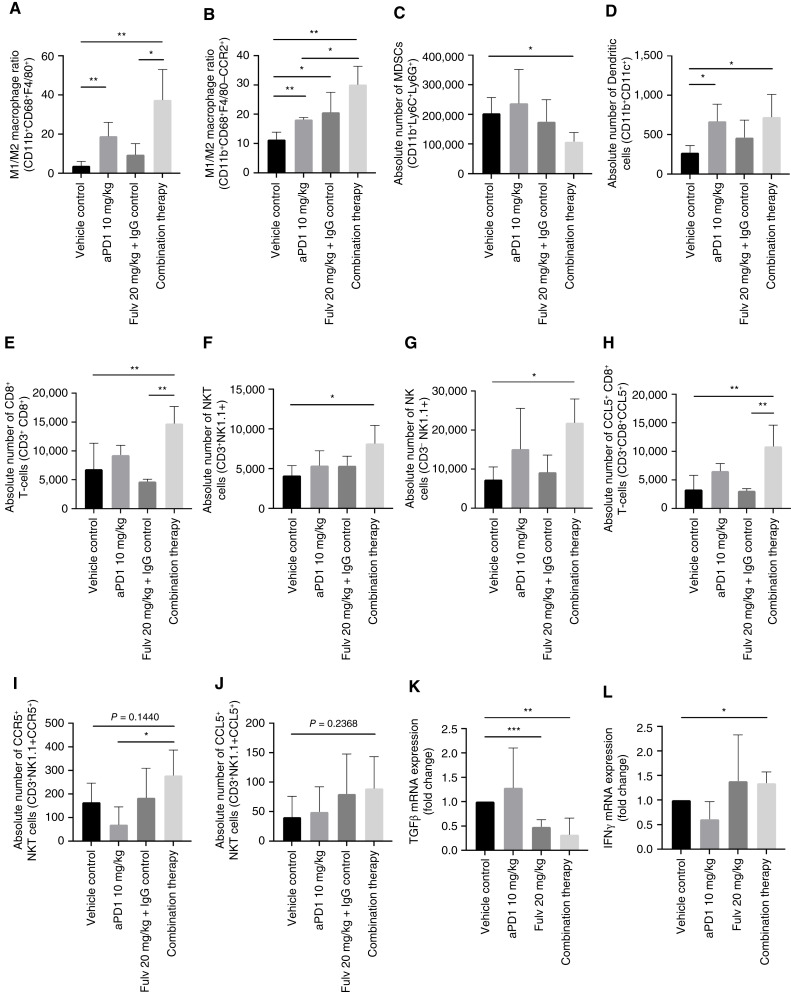
The combinatorial Fulv and anti-PD-1 immunotherapy reprograms the IME of CRCLM. Hepatic immune cells were isolated following the treatment regimen depicted in Fig. 6A. Shown in **A–J** are results of FC performed on cells from the indicated treatment groups (*n* = 4). Shown in **K** and **L** are results of RT-qPCR performed on whole liver RNA (*n* = 3), normalized to GAPDH, and expressed as means (± SD) fold change relative to vehicle (assigned a value of 1). *, *P* ≤ 0.05; **, *P* ≤ 0.01; ***, *P* ≤ 0.001, Student *t* test.

The marked effect of the combinatorial therapy on LM suggested that Fulv treatment may have facilitated a T cells–mediated anti-tumor response in the liver ME. To identify the changes in the TME, we isolated HIC from mice treated as described above for analysis by FC. We found in these mice an increase in M1/M2 macrophage ratio in both the CD11b^+^CD68^+^F4/80^+^ (Fig. 7A) and CD11b^+^CD68^+^CCR2^+^ (Fig. 7B) macrophage subsets, a decrease in CD11b^+^Ly6C^+^Ly6G^+^ MDSC (Fig. 7C) and a higher accumulation of CD11b^+^CD11c^+^ dendritic cells (DC; Fig. 7D), suggesting that the immunosuppressive ME in these mice was reprogramed as a consequence of the treatment. Moreover, a significant increase in CD8^+^ T, NKT, and NK cells was observed in mice treated with the combinatorial therapy relative to controls; an effect specific to this treatment group (Fig. 7E–G respectively) and similarly to our findings in OVX mice, we found that the accumulations of CCL5^+^CD8^+^ T cells (Fig. 7H) and CCR5^+^ NKT cells (Fig. 7I), and to a lesser extent of CCL5^+^ NKT cells (Fig. 7J), in the liver ME were increased in this treatment group relative to all other treatment groups. This suggested that the IME in these livers was reprogrammed to enhance an antitumor immune response. Furthermore, RNA isolated from the livers of these mice and analyzed by RT-qPCR revealed a significant reduction in the expression of immunosuppressive TGF-β (Fig. 7K) accompanied by a significant increase in IFNγ (Fig. 7L) expression levels, suggesting that reprogramming the TIME by Fulv and the PD-1 inhibitor potentiated a cytotoxic response in these livers.

## Discussion

**Figure 8:**
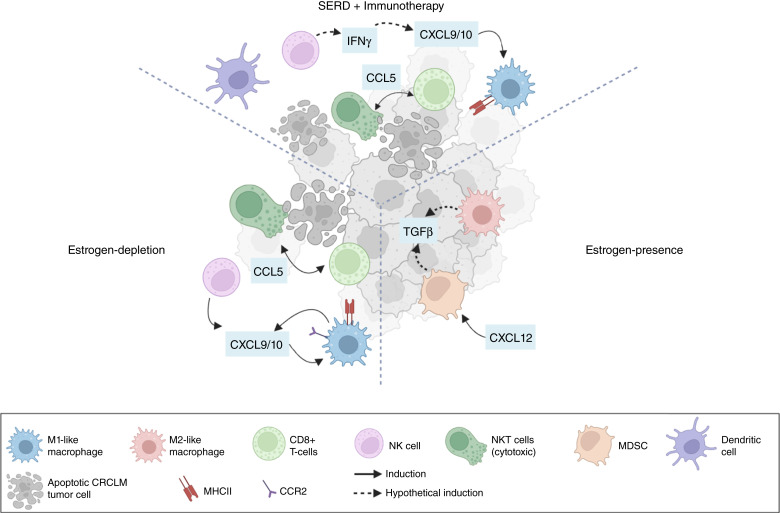
A proposed model for the role of estrogen in the regulation of the TME of CRCLM and the effect of ER blockade by Fulv. The diagram depicts postulated mechanisms for the immunosuppressive role of estrogen in the liver and the effects of its depletion or blockade. ER/E2 signaling can promote CRCLM by regulating the recruitment and activity of innate and adaptive immune cells. Ovariectomy or Fulv treatment result in increased accumulation of NK/NKT cells and M1-like macrophages, increasing the antitumor immune response. Fulv treatment can thereby potentiate the response to immunotherapy and augment the eradication of metastatic tumor cells.

The innate and adaptive immune cells in the TME of the liver, including macrophages, NK, NKT, and CD8^+^ T cells, control anticancer immunity and tumor progression. We observed an increased M1/M2 macrophage ratio in estrogen-depleted mice, particularly in the recruited subset. CXCR3^+^CD68^+^CCR2^+^ macrophages were more prevalent in OVX mice, suggesting that CXCR3 may be involved in maintaining macrophages in the M1 polarization state. Estrogen depletion also enhanced liver NKT cell accumulation and cytotoxicity, and this was reversible with E2 supplementation. Production of the chemokines CCL5/RANTES, CXCL10/IP-19, CXCL9/MIG, and CCL2/MCP1 increased, while TGFβ levels decreased in OVX mice (see summary diagram in Fig. 8).

While the role of the CCL5/CCR5 axis in cancer has been a subject of conflicting reports (35), we found that CRCLM outgrowth increased in OVX mice treated with the CCR5 antagonist Mvc, suggesting a tumor-inhibitory role for CCL5 in this TME. The increase in CCL5^+^/CCR5^+^ CD8^+^T and NKT cells in the livers of OVX mice suggest that estrogen regulates the recruitment of these cells via local CCL5 production. Treatment with Fulv had similar effects on T- and NKT-cell recruitment and caused a reduction in CRCLM that was further augmented with anti-PD-1 therapy, suggesting that ER targeting reprogramed the liver TIME for antitumor responses. Anti-PD-1 alone also moderately (but not significantly) reduced CRCLM, possibly due to MC-38 cell immunogenicity (36).

Previously we have shown that estrogen depletion reduced MDSC accumulation in the liver TME (12). The present study provides additional mechanistic insight by identifying a set of chemokines that are downregulated in estrogen-competent mice and can control immune cell accumulation. Estradiol and the ERs can influence pro-inflammatory cytokine production, in a dose-dependent and a cell and organ context–specific manner (37). ERα can affect cytokine production and immune cell recruitment via direct interaction with NF-κB, as was shown for the suppression of *mcp1* (CCL2) expression (37). Estrogen may also indirectly affect immune cell function via upregulation of immunosuppressive factors such as TGF-β and IL-6 (37, 38). Our data suggest that in the liver TIME, estrogen plays a predominantly immunosuppressive role, dampening antitumor immunity.

The role of estrogen in macrophage polarization is not well understood. In line with our findings, Chakraborty and colleagues (28) reported that ERα promoted M2 polarization in melanoma, suppressing CD8^+^ T-cell responses and contributing to immunotherapy resistance. We found increased M1-like macrophage accumulation in estrogen-depleted mice, where elevated CXCL10 and CCL2 levels were observed, consistent with findings in a pancreatic cancer model where CXCL10/CXCR3 signaling was shown to maintain macrophages in the pro-inflammatory M1 phenotype (39). Moreover, several studies have shown that CCL2 favors pro-inflammatory macrophage polarization (40–43). Thus, the increased CXCL10/CXCR3 signaling in OVX mice may have contributed to preferential accumulation of M1-like macrophages, although a more direct role of estrogen receptor signaling cannot be ruled out (28). Of relevance, single-cell RNA sequencing and spatial transcriptomics recently identified the highly metabolically activated *MRC1*^+^*CCL18*^+^ M2-like macrophages in human CRCLM and their accumulation was shown to correlate with progressive disease (44), supporting their relevance to the human disease.

We previously documented a reduction in G-MDSC recruitment in livers of OVX mice (12). The IME of LM involves complex cell–cell communications (45, 46), but several lines of evidence support a link between the present findings and the reduction in G-MDSC accumulation. The reduction in CXCL12/SDF-1 in OVX mice documented here is in line with its reported role in MDSC migration and survival, the decreased response to anti-PD-1 therapy and increased LM outgrowth (47–49). Additionally, reduced MDSC recruitment in OVX mice may increase CCL5 production in the liver by increasing the numbers of cytotoxic CD8^+^ T and NKT cells—major sources of this chemokine (50). Moreover, the increase in T-cell derived IFNγ observed in OVX mice and in mice treated with Fulv and anti-PD-1 may have contributed to M1-like macrophage polarization and chemokine CCL2 and CXCL10 production (51).

We observed increased NKT-cell cytotoxicity in OVX mice. NKT-cell infiltration was identified as a favorable prognostic factor for overall survival in patients with colorectal carcinoma, suggesting that these cells may impede metastatic outgrowth (52). Moreover, CCL5 expression in T cells was linked to their cytotoxicity in CRCLM and a recent report documented a cooperative interaction between CXCL9/10 and CCL5 in the recruitment of effector T cells into human solid tumors (53, 54). This is in line with our findings in both OVX mice and mice treated with the CCR5 inhibitor Mvc.

Combination SERD and anti-PD-1 therapy significantly reduced CRCLM outgrowth. In the treated mice, CCL5^+^ CD8^+^ T and CCR5^+^ NKT cells, dendritic cell accumulation, and M1/M2 macrophage ratios were all increased, mimicking the results in OVX mice. Of relevance, conventional type 1 dendritic cell (cDC1) accumulation was shown to depend on NK-cell–derived CCL5 in mouse tumor models and their levels also correlated with patient survival in multiple cancer types (55). A Swedish study reported that approximately 15% of female patients with LM were under the age of 50 years with CRCLM comprising 21.2% of patients in this group (56). Taken together, our findings suggest that combining SERDs with immunotherapy may benefit premenopausal CRC patients and others with hormone-independent liver-metastatic tumors. Although premenopausal females represent a fraction of the total CRC patient population, this approach warrants consideration, given the aggressive nature of the disease in the younger patients, their resistance to immunotherapy and the availability of FDA-approved anti-estrogenic drugs for disease management.

## Supplementary Material

Figure S1 NK cells cytotoxicity is not significantly different between estrogen-depleted and estrogen-competent mice. NK cells were sorted based on CD3 (PE) and NK1.1 (APC), and MC-38 were pre-incubated with the Incucyte Cytotox Green Dye (Excitation Max 491 nm, Emission Max 509 nm). The NK cells and MC-38 cells were co-cultured at a ratio of 5:1 for up to 30h. Shown in the figure is quantification of dead (green) MC-38 cells normalized to time 0h (n=3).

Figure S2 NKT and CD8+ T cells analyses. Shown in (a) are the multiplex cytokine array results validation at the mRNA expression. Shown in (b) are the flow cytometry gating strategy for the analysis of CCL5 and CCR5 in NKT and CD8+ T cells (n=3-4).

Figure S3 In situ hybridization by RNAscope controls. Shown in the top panels are the RNAscope positive and negative controls, shown in the bottom panels are the immunofluorescence staining for the CD3 antibody mouse IgG1 isotype control and the donkey anti-mouse AF488 secondary antibody alone.

Figure S4 Lymphocytes flow cytometry analyses in FC1199 tumor model. Shown are the lymphocytes flow cytometry analysis of lymphocytes in FC1199 pancreatic liver metastasis model at Day 8 post-tumor injection in SHAM-control, OVX, and OVX + E2 mice (n=4).

Figure S5 Macrophages analyses. Shown in (a) are the M1:M2 macrophages ratio analysis results using additional CD38 marker (n=3-4) and (b) TNF-alpha for M1-like macrophages characterization, and the use of the additional OVX+E2 experimental group which shows a reverse trend upon estrogen supplementation compared to the OVX group (n=3-4). Shown in (c) are the macrophages flow cytometry analysis results in FC1199 pancreatic liver metastasis model at Day 12 post-tumor injection (n=4).

Table S1 Immunofluorescence staining antibodies.

Table S2 Flow cytometry antibodies.

Table S3 qPCR primer sequences.

## References

[1] Zhou H , Liu Z, Wang Y, Wen X, Amador EH, Yuan L, . Colorectal liver metastasis: molecular mechanism and interventional therapy. Signal Transduct Target Ther 2022;7:70. 35246503 10.1038/s41392-022-00922-2 PMC8897452

[2] Siegel RL , Jakubowski CD, Fedewa SA, Davis A, Azad NS. Colorectal cancer in the young: epidemiology, prevention, management. Am Soc Clin Oncol Educ Book 2020;40:1–14. 10.1200/EDBK_279901 32315236

[3] Wu Y , Xu W, Wang L. IDDF2021-ABS-0191 gender matters: sex disparities in colorectal cancer liver metastasis survival: a population-based study. Gut 2021;70:A142–3.

[4] Miller KD , Nogueira L, Mariotto AB, Rowland JH, Yabroff KR, Alfano CM, . Cancer treatment and survivorship statistics, 2019. CA Cancer J Clin 2019;69:363–85. 31184787 10.3322/caac.21565

[5] Siegel R , Naishadham D, Jemal A. Cancer statistics, 2012. CA Cancer J Clin 2012;62:10–29. 22237781 10.3322/caac.20138

[6] Tauriello DVF , Calon A, Lonardo E, Batlle E. Determinants of metastatic competency in colorectal cancer. Mol Oncol 2017;11:97–119. 28085225 10.1002/1878-0261.12018 PMC5423222

[7] Gal-Oz ST , Shay T. Immune sexual dimorphism: connecting the dots. Physiology (Bethesda) 2022;37:55–68. 34514870 10.1152/physiol.00006.2021 PMC8873034

[8] Ye Y , Jing Y, Li L, Mills GB, Diao L, Liu H, . Sex-associated molecular differences for cancer immunotherapy. Nat Commun 2020;11:1779. 32286310 10.1038/s41467-020-15679-x PMC7156379

[9] Cortese N , Marchesi F. Liver metastases “siphon” off immunotherapy response. Hepatobiliary Surg Nutr 2021;10:526–9. 34430535 10.21037/hbsn-21-215 PMC8351020

[10] Zhang Y , Zhao Y, Li Q, Wang Y. Macrophages, as a promising strategy to targeted treatment for colorectal cancer metastasis in tumor immune microenvironment. Front Immunol 2021;12:685978. 34326840 10.3389/fimmu.2021.685978 PMC8313969

[11] Boutilier AJ , Elsawa SF. Macrophage polarization states in the tumor microenvironment. Int J Mol Sci 2021;22:6995. 34209703 10.3390/ijms22136995 PMC8268869

[12] Milette S , Hashimoto M, Perrino S, Qi S, Chen M, Ham B, . Sexual dimorphism and the role of estrogen in the immune microenvironment of liver metastases. Nat Commun 2019;10:5745. 31848339 10.1038/s41467-019-13571-x PMC6917725

[13] Didion JP , Buus RJ, Naghashfar Z, Threadgill DW, Morse HC 3rd, de Villena FP-M. SNP array profiling of mouse cell lines identifies their strains of origin and reveals cross-contamination and widespread aneuploidy. BMC Genomics 2014;15:847. 25277546 10.1186/1471-2164-15-847 PMC4198738

[14] Hingorani SR , Wang L, Multani AS, Combs C, Deramaudt TB, Hruban RH, . Trp53R172H and KrasG12D cooperate to promote chromosomal instability and widely metastatic pancreatic ductal adenocarcinoma in mice. Cancer Cell 2005;7:469–83. 15894267 10.1016/j.ccr.2005.04.023

[15] Wang H , Yin S. Natural killer T cells in liver injury, inflammation and cancer. Expert Rev Gastroenterol Hepatol 2015;9:1077–85. 26068039 10.1586/17474124.2015.1056738

[16] Geissmann F , Cameron TO, Sidobre S, Manlongat N, Kronenberg M, Briskin MJ, . Intravascular immune surveillance by CXCR6+ NKT cells patrolling liver sinusoids. PLoS Biol 2005;3:e113. 15799695 10.1371/journal.pbio.0030113 PMC1073691

[17] Bandyopadhyay K , Marrero I, Kumar V. NKT cell subsets as key participants in liver physiology and pathology. Cell Mol Immunol 2016;13:337–46. 26972772 10.1038/cmi.2015.115 PMC4856801

[18] Webb TJ , Yuan W, Meyer E, Dellabona P. Editorial: NKT cells in cancer immunotherapy. Front Immunol 2020;11:1314. 32655576 10.3389/fimmu.2020.01314 PMC7324679

[19] Elsawa SF , Novak AJ, Ziesmer SC, Almada LL, Hodge LS, Grote DM, . Comprehensive analysis of tumor microenvironment cytokines in Waldenstrom macroglobulinemia identifies CCL5 as a novel modulator of IL-6 activity. Blood 2011;118:5540–9. 21921047 10.1182/blood-2011-04-351742 PMC3217355

[20] Karlmark KR , Wasmuth HE, Trautwein C, Tacke F. Chemokine-directed immune cell infiltration in acute and chronic liver disease. Expert Rev Gastroenterol Hepatol 2008;2:233–42. 19072358 10.1586/17474124.2.2.233

[21] Tarique AA , Logan J, Thomas E, Holt PG, Sly PD, Fantino E. Phenotypic, functional, and plasticity features of classical and alternatively activated human macrophages. Am J Respir Cell Mol Biol 2015;53:676–88. 25870903 10.1165/rcmb.2015-0012OC

[22] Woollard SM , Kanmogne GD. Maraviroc: a review of its use in HIV infection and beyond. Drug Des Devel Ther 2015;9:5447–68. 10.2147/DDDT.S90580 PMC4598208 26491256

[23] Antonelli A , Ferrari SM, Giuggioli D, Ferrannini E, Ferri C, Fallahi P. Chemokine (C-X-C motif) ligand (CXCL)10 in autoimmune diseases. Autoimmun Rev 2014;13:272–80. 24189283 10.1016/j.autrev.2013.10.010

[24] Wang X , Zhang Y, Wang S, Ni H, Zhao P, Chen G, . The role of CXCR3 and its ligands in cancer. Front Oncol 2022;12:1022688. 36479091 10.3389/fonc.2022.1022688 PMC9720144

[25] Marra F , Tacke F. Roles for chemokines in liver disease. Gastroenterology 2014;147:577–94.e1. 25066692 10.1053/j.gastro.2014.06.043

[26] Yan Y , Zheng L, Du Q, Yazdani H, Dong K, Guo Y, . Interferon regulatory factor 1(IRF-1) activates anti-tumor immunity via CXCL10/CXCR3 axis in hepatocellular carcinoma (HCC). Cancer Lett 2021;506:95–106. 33689775 10.1016/j.canlet.2021.03.002 PMC8009854

[27] Klein I , Cornejo JC, Polakos NK, John B, Wuensch SA, Topham DJ, . Kupffer cell heterogeneity: functional properties of bone marrow–derived and sessile hepatic macrophages. Blood 2007;110:4077–85. 17690256 10.1182/blood-2007-02-073841 PMC2190614

[28] Chakraborty B , Byemerwa J, Shepherd J, Haines CN, Baldi R, Gong W, . Inhibition of estrogen signaling in myeloid cells increases tumor immunity in melanoma. J Clin Invest 2021;131:e151347. 34637400 10.1172/JCI151347 PMC8631601

[29] Ahmed I , Ismail N. M1 and M2 macrophages polarization via mTORC1 influences innate immunity and outcome of *Ehrlichia* infection. J Cell Immunol 2020;2:108–15. 32719831 10.33696/immunology.2.029 PMC7384756

[30] Rocca A , Maltoni R, Bravaccini S, Donati C, Andreis D. Clinical utility of fulvestrant in the treatment of breast cancer: a report on the emerging clinical evidence. Cancer Manag Res 2018;10:3083–99. 30214302 10.2147/CMAR.S137772 PMC6124791

[31] Boér K . Fulvestrant in advanced breast cancer: evidence to date and place in therapy. Ther Adv Med Oncol 2017;9:465–79. 28717399 10.1177/1758834017711097 PMC5502950

[32] Varayathu H , Sarathy V, Thomas BE, Mufti SS, Naik R. Combination strategies to augment immune check point inhibitors efficacy - implications for translational research. Front Oncol 2021;11:559161. 34123767 10.3389/fonc.2021.559161 PMC8193928

[33] Tumeh PC , Hellmann MD, Hamid O, Tsai KK, Loo KL, Gubens MA, . Liver metastasis and treatment outcome with anti-PD-1 monoclonal antibody in patients with melanoma and NSCLC. Cancer Immunol Res 2017;5:417–24. 28411193 10.1158/2326-6066.CIR-16-0325 PMC5749922

[34] Rothenberger NJ , Somasundaram A, Stabile LP. The role of the estrogen pathway in the tumor microenvironment. Int J Mol Sci 2018;19:611. 29463044 10.3390/ijms19020611 PMC5855833

[35] Aldinucci D , Borghese C, Casagrande N. The CCL5/CCR5 axis in cancer progression. Cancers (Basel) 2020;12:1765. 32630699 10.3390/cancers12071765 PMC7407580

[36] Efremova M , Rieder D, Klepsch V, Charoentong P, Finotello F, Hackl H, . Targeting immune checkpoints potentiates immunoediting and changes the dynamics of tumor evolution. Nat Commun 2018;9:32. 29296022 10.1038/s41467-017-02424-0 PMC5750210

[37] Kovats S . Estrogen receptors regulate innate immune cells and signaling pathways. Cell Immunol 2015;294:63–9. 25682174 10.1016/j.cellimm.2015.01.018 PMC4380804

[38] Hawse JR , Subramaniam M, Ingle JN, Oursler MJ, Rajamannan NM, Spelsberg TC. Estrogen-TGFβ cross-talk in bone and other cell types: role of TIEG, Runx2, and other transcription factors. J Cell Biochem 2008;103:383–92. 17541956 10.1002/jcb.21425 PMC3372922

[39] Pandey V , Fleming-Martinez A, Bastea L, Doeppler HR, Eisenhauer J, Le T, . CXCL10/CXCR3 signaling contributes to an inflammatory microenvironment and its blockade enhances progression of murine pancreatic precancerous lesions. Elife 2021;10:e60646. 34328416 10.7554/eLife.60646 PMC8360647

[40] Wang Q , Ren J, Morgan S, Liu Z, Dou C, Liu B. Monocyte chemoattractant protein-1 (MCP-1) regulates macrophage cytotoxicity in abdominal aortic aneurysm. PLoS One 2014;9:e92053. 24632850 10.1371/journal.pone.0092053 PMC3954911

[41] Sodhi A , Biswas SK. Monocyte chemoattractant protein-1-induced activation of p42/44 MAPK and c-Jun in murine peritoneal macrophages: a potential pathway for macrophage activation. J Interferon Cytokine Res 2002;22:517–26. 12060490 10.1089/10799900252981990

[42] Mu J . RhoA signaling in CCL2-induced macrophage polarization. J Allergy Clin Immunol 2018;141:AB114.

[43] Nio Y , Yamauchi T, Iwabu M, Okada-Iwabu M, Funata M, Yamaguchi M, . Monocyte chemoattractant protein-1 (MCP-1) deficiency enhances alternatively activated M2 macrophages and ameliorates insulin resistance and fatty liver in lipoatrophic diabetic A-ZIP transgenic mice. Diabetologia 2012;55:3350–8. 22983634 10.1007/s00125-012-2710-2

[44] Wu Y , Yang S, Ma J, Chen Z, Song G, Rao D, . Spatiotemporal immune landscape of colorectal cancer liver metastasis at single-cell level. Cancer Discov 2022;12:134–53. 34417225 10.1158/2159-8290.CD-21-0316

[45] Ciner AT , Jones K, Muschel RJ, Brodt P. The unique immune microenvironment of liver metastases: challenges and opportunities. Semin Cancer Biol 2021;71:143–56. 32526354 10.1016/j.semcancer.2020.06.003

[46] Tsilimigras DI , Brodt P, Clavien PA, Muschel RJ, D’Angelica MI, Endo I, . Liver metastases. Nat Rev Dis Primers 2021;7:27. 33859205 10.1038/s41572-021-00261-6

[47] Jiang K , Li J, Zhang J, Wang L, Zhang Q, Ge J, . SDF-1/CXCR4 axis facilitates myeloid-derived suppressor cells accumulation in osteosarcoma microenvironment and blunts the response to anti-PD-1 therapy. Int Immunopharmacol 2019;75:105818. 31437795 10.1016/j.intimp.2019.105818

[48] Xu Y , Fang F, Jiao H, Zheng X, Huang L, Yi X, . Activated hepatic stellate cells regulate MDSC migration through the SDF-1/CXCR4 axis in an orthotopic mouse model of hepatocellular carcinoma. Cancer Immunol Immunother 2019;68:1959–69. 31641797 10.1007/s00262-019-02414-9 PMC11028284

[49] Benedicto A , Romayor I, Arteta B. CXCR4 receptor blockage reduces the contribution of tumor and stromal cells to the metastatic growth in the liver. Oncol Rep 2018;39:2022–30. 29436696 10.3892/or.2018.6254

[50] Zeng Z , Lan T, Wei Y, Wei X. CCL5/CCR5 axis in human diseases and related treatments. Genes Dis 2022;9:12–27. 34514075 10.1016/j.gendis.2021.08.004 PMC8423937

[51] Ivashkiv LB . IFNγ: signalling, epigenetics and roles in immunity, metabolism, disease and cancer immunotherapy. Nat Rev Immunol 2018;18:545–58. 29921905 10.1038/s41577-018-0029-z PMC6340644

[52] Tachibana T , Onodera H, Tsuruyama T, Mori A, Nagayama S, Hiai H, . Increased intratumor Valpha24-positive natural killer T cells: a prognostic factor for primary colorectal carcinomas. Clin Cancer Res 2005;11:7322–7. 16243803 10.1158/1078-0432.CCR-05-0877

[53] Massalha H , Bahar Halpern K, Abu-Gazala S, Jana T, Massasa EE, Moor AE, . A single cell atlas of the human liver tumor microenvironment. Mol Syst Biol 2020;16:e9682. 33332768 10.15252/msb.20209682 PMC7746227

[54] Dangaj D , Bruand M, Grimm AJ, Ronet C, Barras D, Duttagupta PA, . Cooperation between constitutive and inducible chemokines enables T cell engraftment and immune attack in solid tumors. Cancer Cell 2019;35:885–900.e10. 31185212 10.1016/j.ccell.2019.05.004 PMC6961655

[55] Böttcher JP , Bonavita E, Chakravarty P, Blees H, Cabeza-Cabrerizo M, Sammicheli S, . NK cells stimulate recruitment of cDC1 into the tumor microenvironment promoting cancer immune control. Cell 2018;172:1022–37.e14. 29429633 10.1016/j.cell.2018.01.004 PMC5847168

[56] de Ridder J , de Wilt JHW, Simmer F, Overbeek L, Lemmens V, Nagtegaal I. Incidence and origin of histologically confirmed liver metastases: an explorative case-study of 23,154 patients. Oncotarget 2016;7:55368–76. 27421135 10.18632/oncotarget.10552 PMC5342423

