# Supplementary material for: Estrogen Receptor Blockade Potentiates Immunotherapy for Liver Metastases by Altering the Liver Immunosuppressive Microenvironment
Source: Cancer Res Commun. 2024 Aug 8;4(8):1963–77. doi: 10.1158/2767-9764.CRC-24-0196 (PMC11306998; doi:10.1158/2767-9764.CRC-24-0196)
Supplement: Figure S1 — NK cells cytotoxicity is not significantly different between estrogen-depleted and estrogen-competent mice. NK cells were sorted based on CD3 (PE) and NK1.1 (APC), and MC-38 were pre-incubated with the Incucyte Cytotox Green Dye (Excitation Max 491 nm, Emission Max 509 nm). The NK cells and MC-38 cells were co-cultured at a ratio of 5:1 for up to 30h. Shown in the figure is quantification of dead (green) MC-38 cells normalized to time 0h (n=3). [file crc-24-0196_figure_s1_supps1.pptx]

## Slide 1
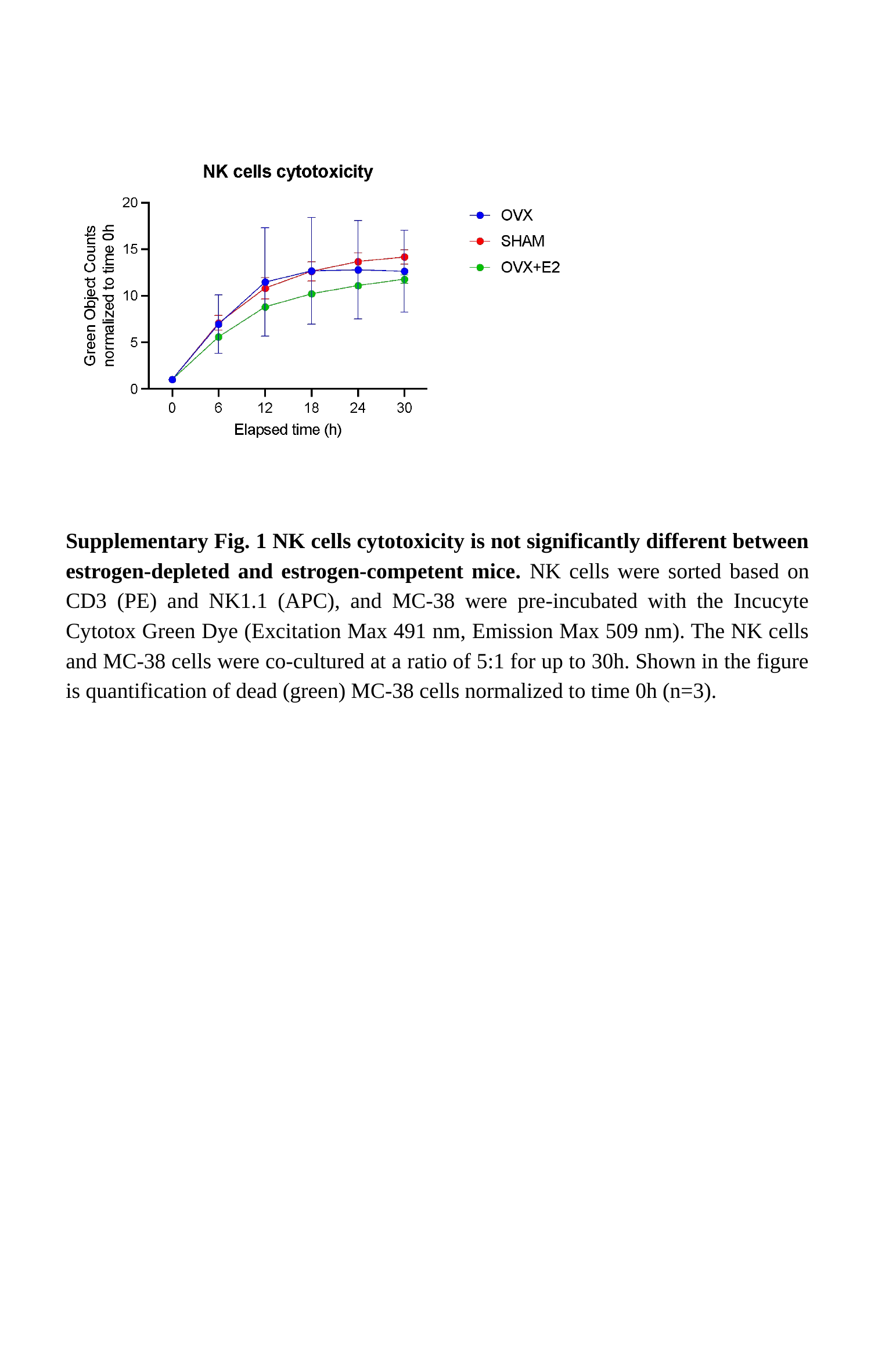

Supplementary Fig. 1 NK cells cytotoxicity is not significantly different between estrogen-depleted and estrogen-competent mice. NK cells were sorted based on CD3 (PE) and NK1.1 (APC), and MC-38 were pre-incubated with the Incucyte Cytotox Green Dye (Excitation Max 491 nm, Emission Max 509 nm). The NK cells and MC-38 cells were co-cultured at a ratio of 5:1 for up to 30h. Shown in the figure is quantification of dead (green) MC-38 cells normalized to time 0h (n=3).
