# Supplementary material for: Estrogen Receptor Blockade Potentiates Immunotherapy for Liver Metastases by Altering the Liver Immunosuppressive Microenvironment
Source: Cancer Res Commun. 2024 Aug 8;4(8):1963–77. doi: 10.1158/2767-9764.CRC-24-0196 (PMC11306998; doi:10.1158/2767-9764.CRC-24-0196)
Supplement: Figure S2 — NKT and CD8+ T cells analyses. Shown in (a) are the multiplex cytokine array results validation at the mRNA expression. Shown in (b) are the flow cytometry gating strategy for the analysis of CCL5 and CCR5 in NKT and CD8+ T cells (n=3-4). [file crc-24-0196_figure_s2_supps2.pptx]

## Slide 1
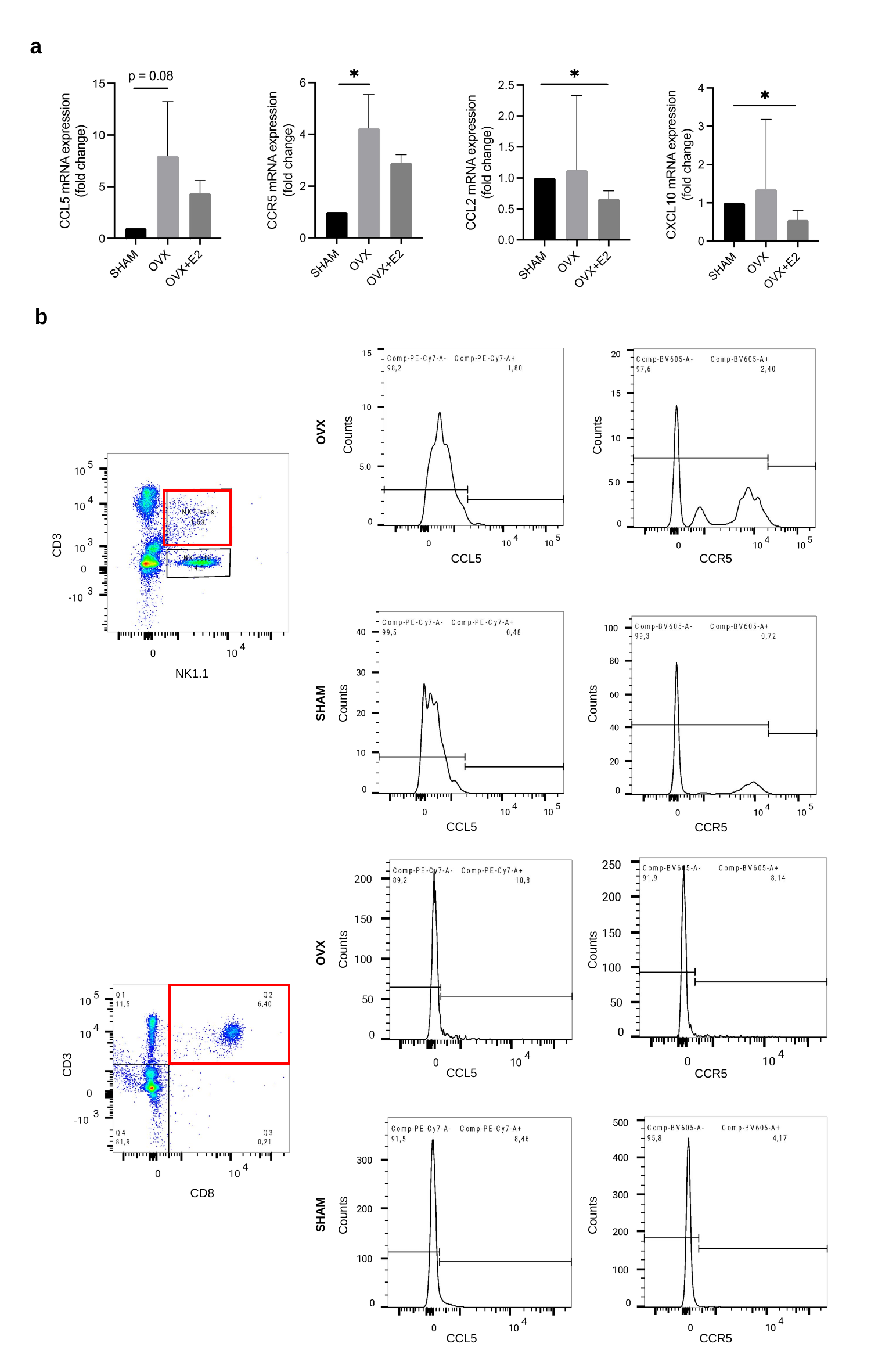

a
b
OVX
Counts
Counts
CD3
CCL5
CCR5
NK1.1
Counts
SHAM
Counts
CCL5
CCR5
Counts
Counts
OVX
CD3
CCL5
CCR5
CD8
SHAM
Counts
Counts
CCL5
CCR5

## Slide 2
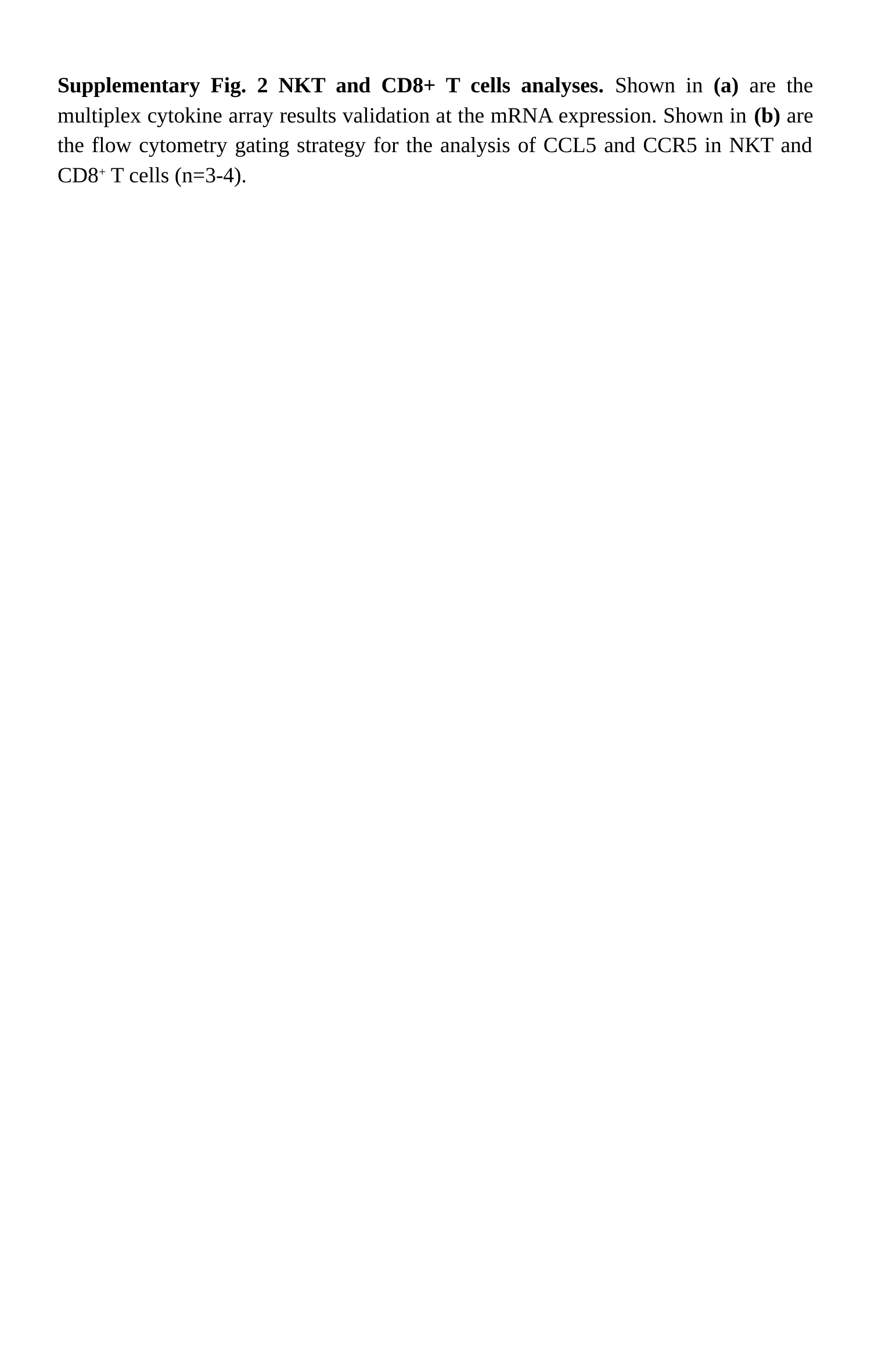

Supplementary Fig. 2 NKT and CD8+ T cells analyses. Shown in (a) are the multiplex cytokine array results validation at the mRNA expression. Shown in (b) are the flow cytometry gating strategy for the analysis of CCL5 and CCR5 in NKT and CD8+ T cells (n=3-4).
