# Supplementary material for: Estrogen Receptor Blockade Potentiates Immunotherapy for Liver Metastases by Altering the Liver Immunosuppressive Microenvironment
Source: Cancer Res Commun. 2024 Aug 8;4(8):1963–77. doi: 10.1158/2767-9764.CRC-24-0196 (PMC11306998; doi:10.1158/2767-9764.CRC-24-0196)
Supplement: Figure S3 — In situ hybridization by RNAscope controls. Shown in the top panels are the RNAscope positive and negative controls, shown in the bottom panels are the immunofluorescence staining for the CD3 antibody mouse IgG1 isotype control and the donkey anti-mouse AF488 secondary antibody alone. [file crc-24-0196_figure_s3_supps3.pptx]

## Slide 1
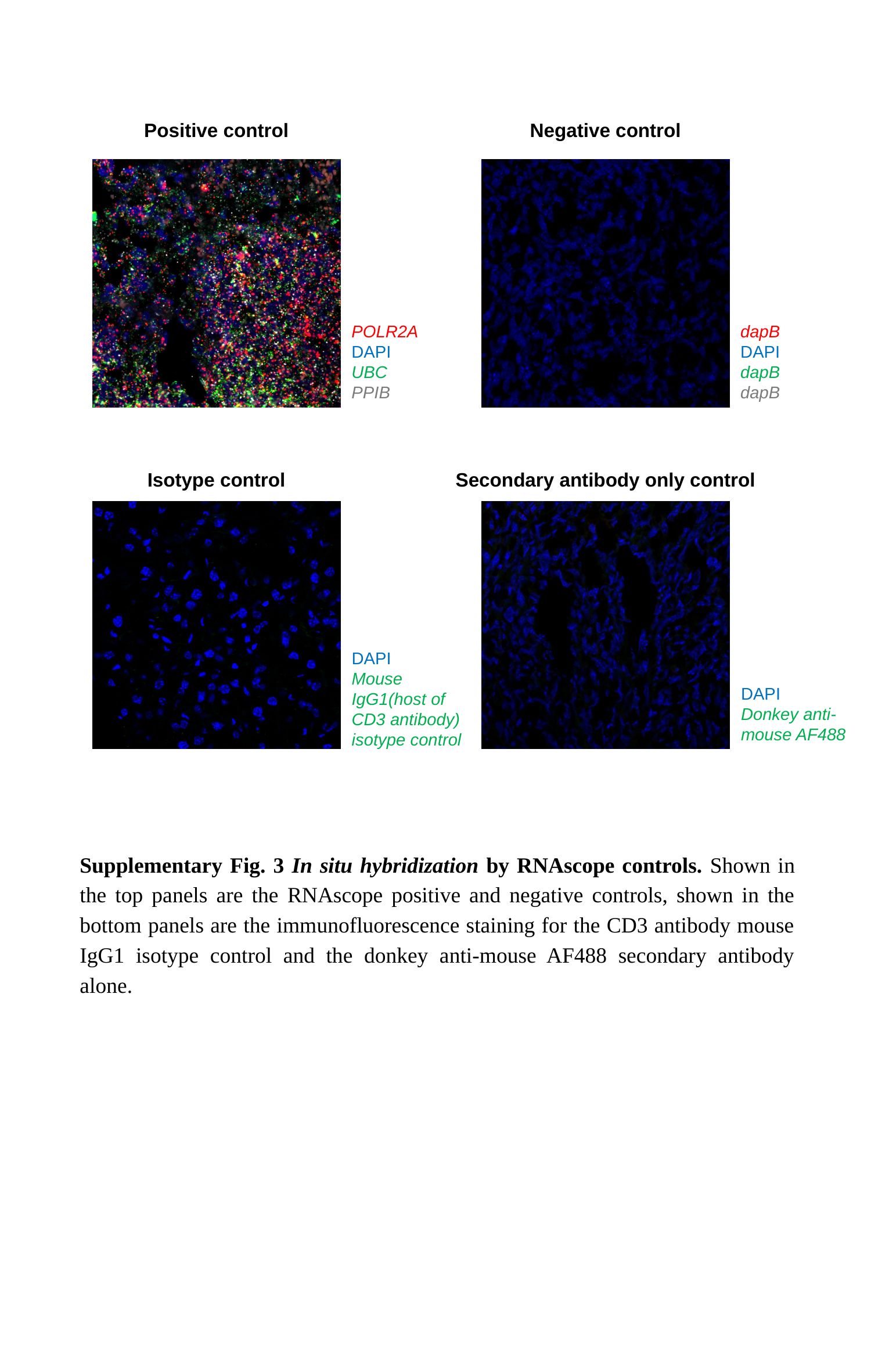

Positive control
Negative control
POLR2A
DAPI
UBC
PPIB
dapB
DAPI
dapB
dapB
Isotype control
Secondary antibody only control
DAPI
Mouse IgG1(host of CD3 antibody) isotype control
DAPI
Donkey anti-mouse AF488
Supplementary Fig. 3 In situ hybridization by RNAscope controls. Shown in the top panels are the RNAscope positive and negative controls, shown in the bottom panels are the immunofluorescence staining for the CD3 antibody mouse IgG1 isotype control and the donkey anti-mouse AF488 secondary antibody alone.
