# Supplementary material for: Estrogen Receptor Blockade Potentiates Immunotherapy for Liver Metastases by Altering the Liver Immunosuppressive Microenvironment
Source: Cancer Res Commun. 2024 Aug 8;4(8):1963–77. doi: 10.1158/2767-9764.CRC-24-0196 (PMC11306998; doi:10.1158/2767-9764.CRC-24-0196)
Supplement: Figure S4 — Lymphocytes flow cytometry analyses in FC1199 tumor model. Shown are the lymphocytes flow cytometry analysis of lymphocytes in FC1199 pancreatic liver metastasis model at Day 8 post-tumor injection in SHAM-control, OVX, and OVX + E2 mice (n=4). [file crc-24-0196_figure_s4_supps4.pptx]

## Slide 1
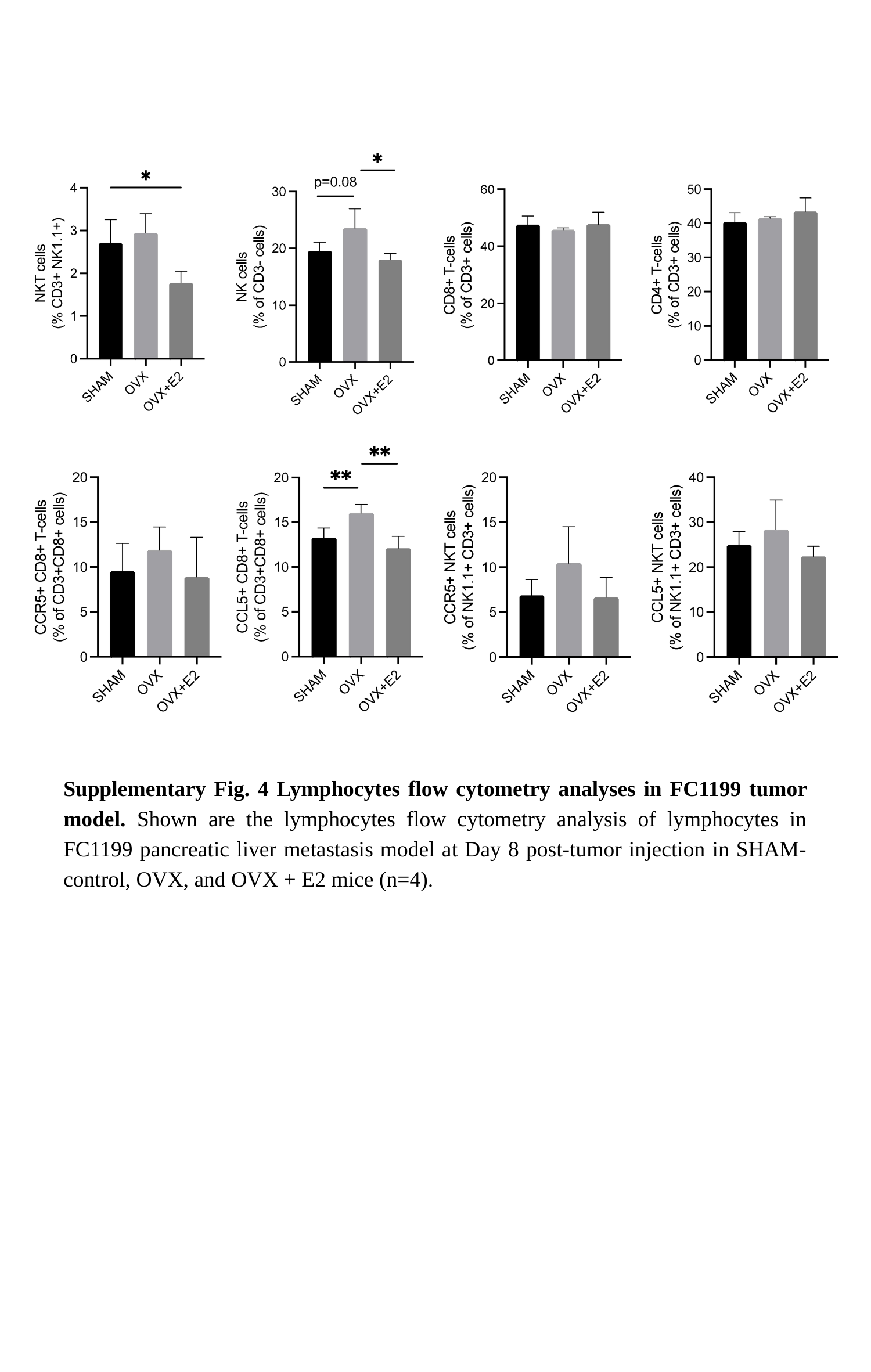

Supplementary Fig. 4 Lymphocytes flow cytometry analyses in FC1199 tumor model. Shown are the lymphocytes flow cytometry analysis of lymphocytes in FC1199 pancreatic liver metastasis model at Day 8 post-tumor injection in SHAM-control, OVX, and OVX + E2 mice (n=4).
