# Supplementary material for: Estrogen Receptor Blockade Potentiates Immunotherapy for Liver Metastases by Altering the Liver Immunosuppressive Microenvironment
Source: Cancer Res Commun. 2024 Aug 8;4(8):1963–77. doi: 10.1158/2767-9764.CRC-24-0196 (PMC11306998; doi:10.1158/2767-9764.CRC-24-0196)
Supplement: Figure S5 — Macrophages analyses. Shown in (a) are the M1:M2 macrophages ratio analysis results using additional CD38 marker (n=3-4) and (b) TNF-alpha for M1-like macrophages characterization, and the use of the additional OVX+E2 experimental group which shows a reverse trend upon estrogen supplementation compared to the OVX group (n=3-4). Shown in (c) are the macrophages flow cytometry analysis results in FC1199 pancreatic liver metastasis model at Day 12 post-tumor injection (n=4). [file crc-24-0196_figure_s5_supps5.pptx]

## Slide 1
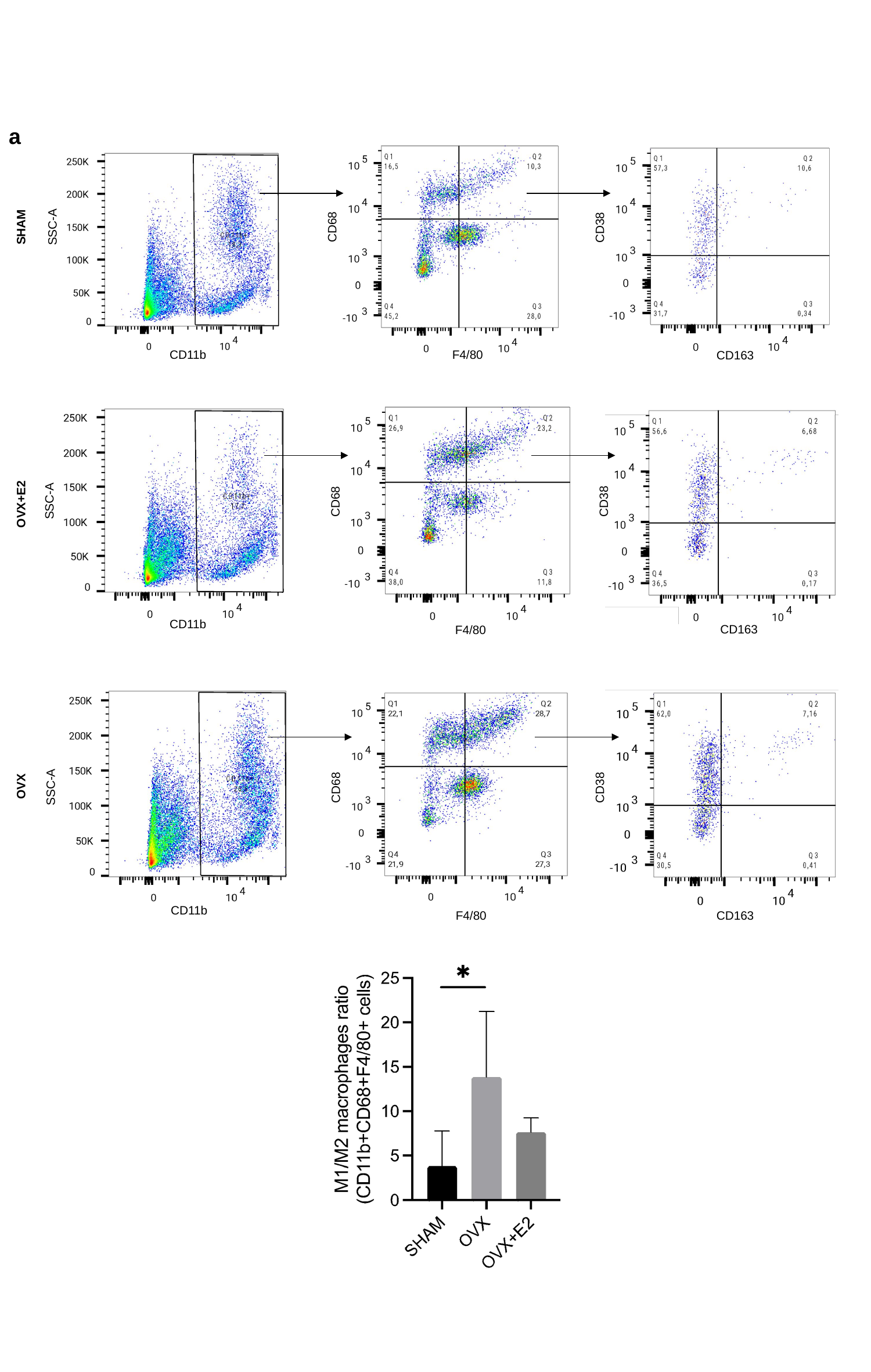

a
CD68
SSC-A
SHAM
CD38
F4/80
CD11b
CD163
SSC-A
CD38
CD68
OVX+E2
CD11b
CD163
F4/80
OVX
SSC-A
CD68
CD38
CD11b
F4/80
CD163

## Slide 2
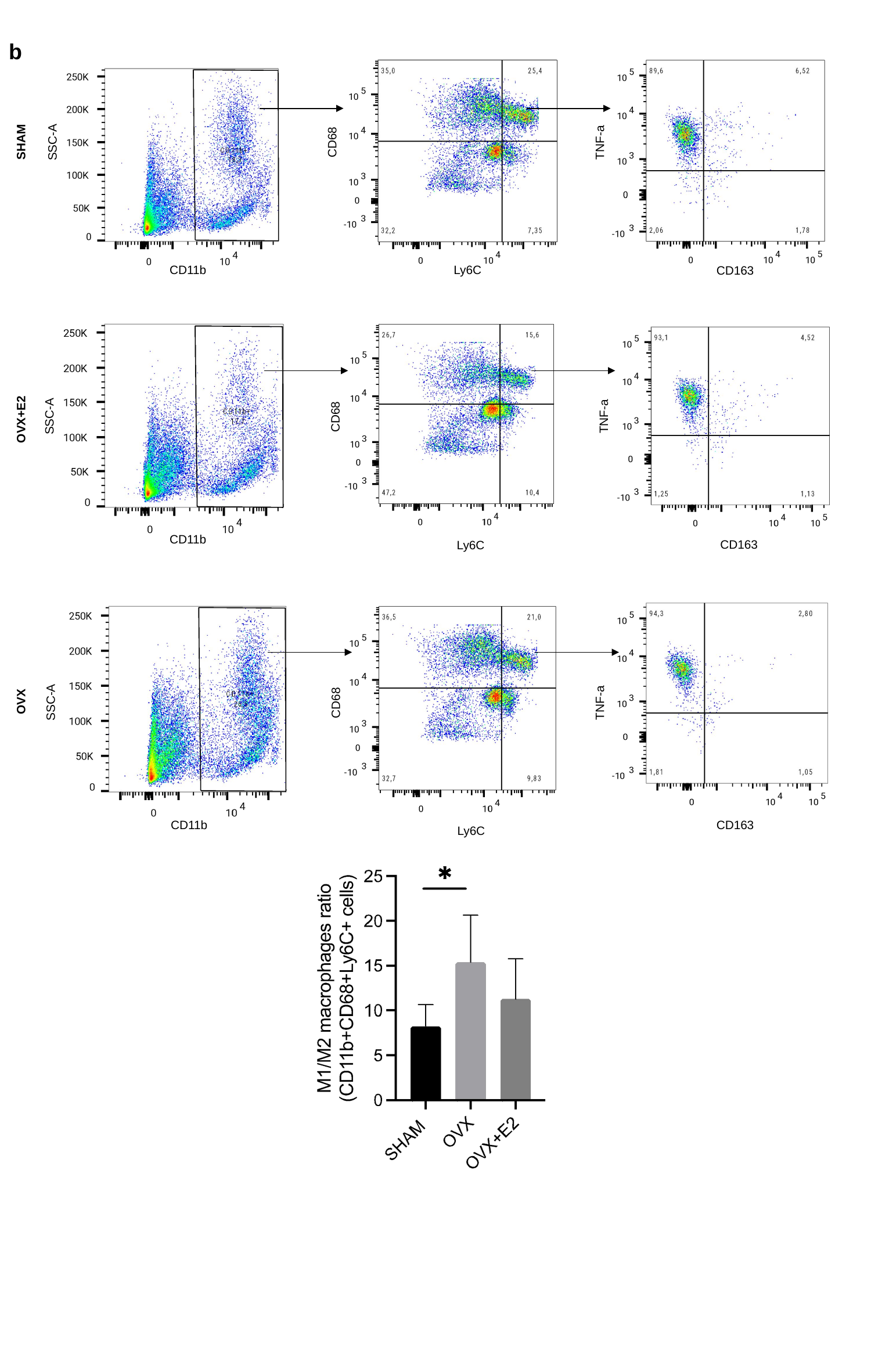

b
CD68
SSC-A
SHAM
TNF-a
Ly6C
CD11b
CD163
SSC-A
TNF-a
CD68
OVX+E2
CD11b
CD163
Ly6C
OVX
SSC-A
CD68
TNF-a
CD11b
CD163
Ly6C

## Slide 3
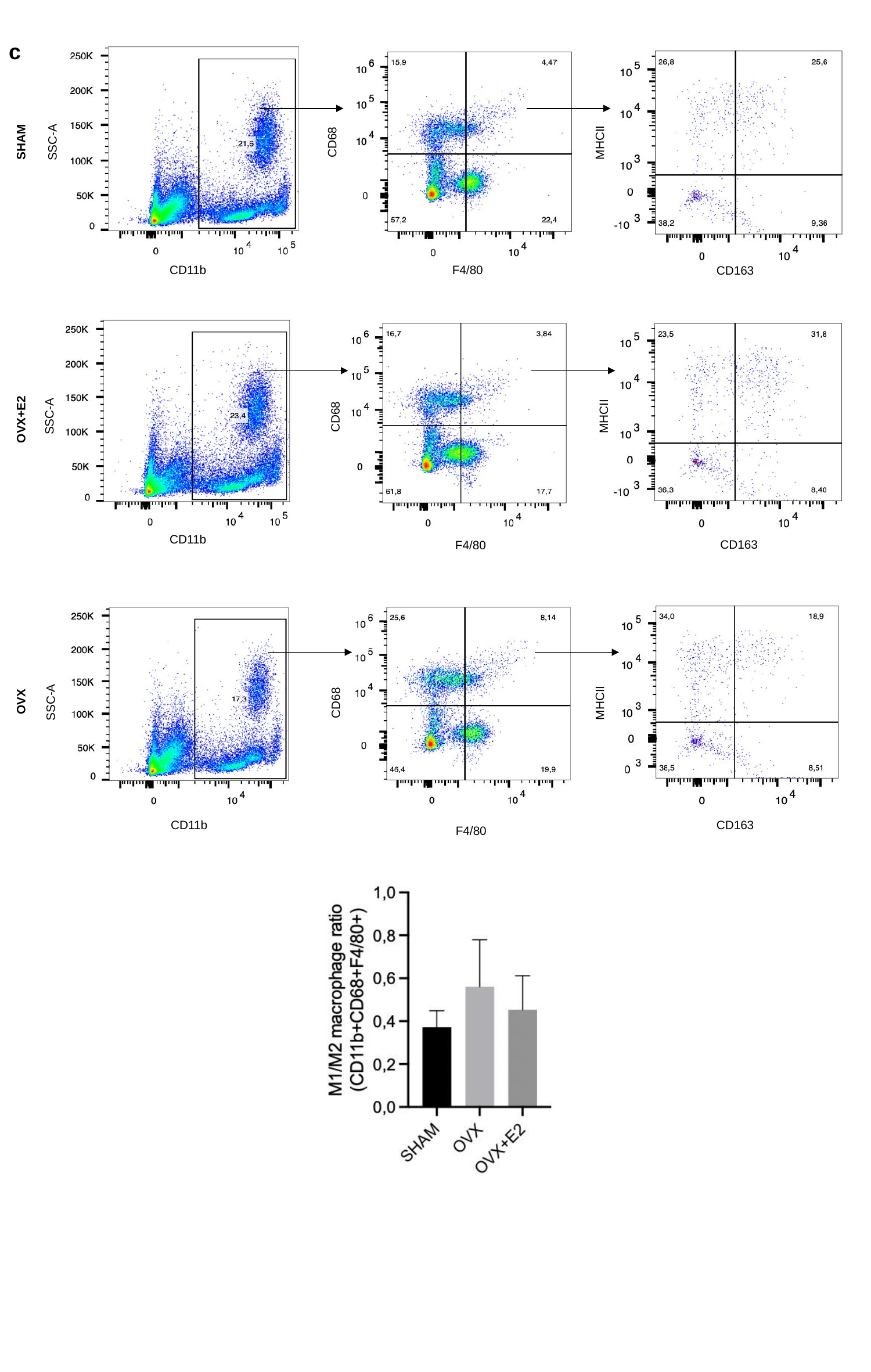

c
CD68
SSC-A
SHAM
MHCII
F4/80
CD11b
CD163
SSC-A
MHCII
CD68
OVX+E2
CD11b
CD163
F4/80
OVX
SSC-A
CD68
MHCII
CD11b
CD163
F4/80

## Slide 4
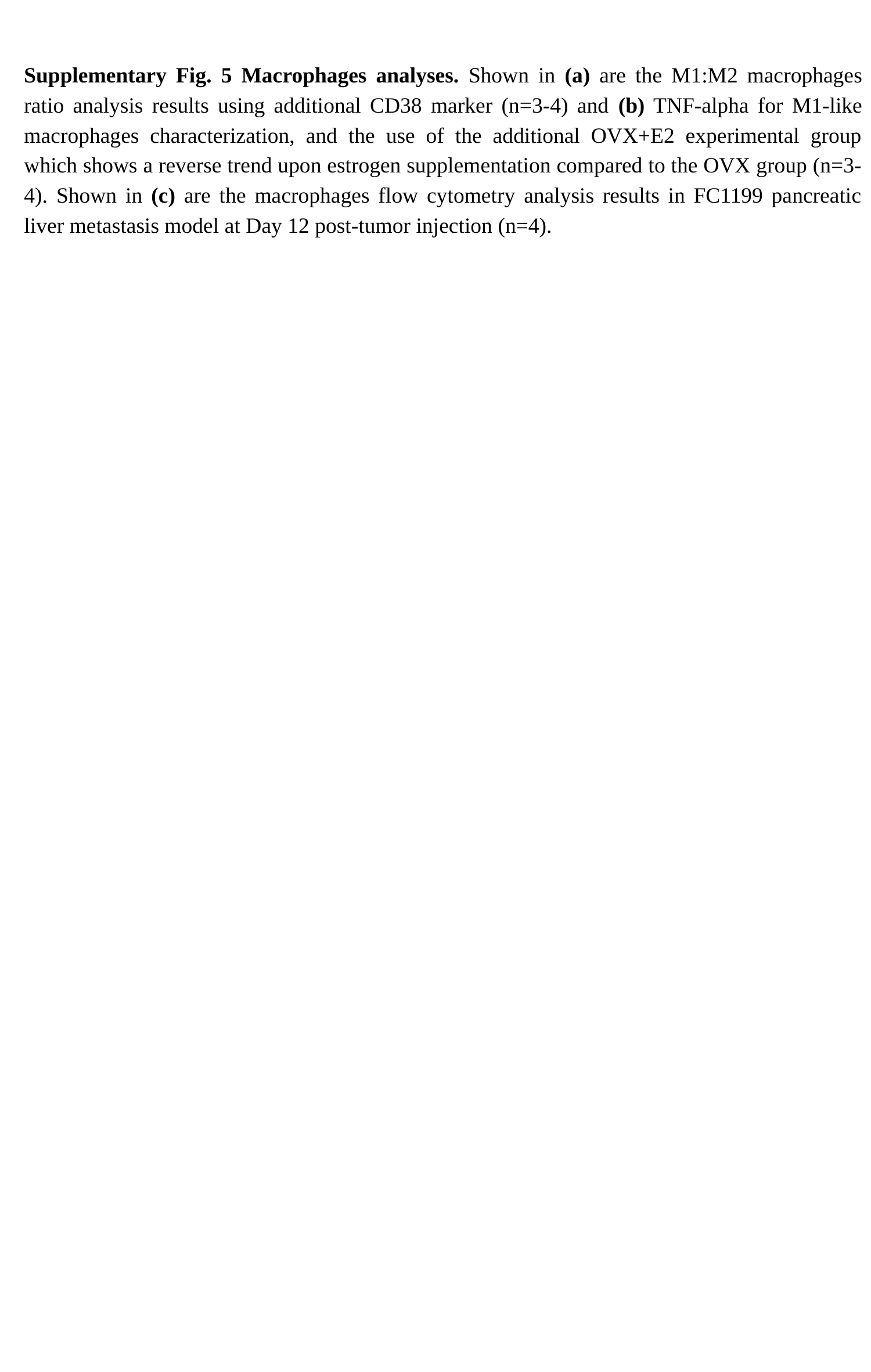

Supplementary Fig. 5 Macrophages analyses. Shown in (a) are the M1:M2 macrophages ratio analysis results using additional CD38 marker (n=3-4) and (b) TNF-alpha for M1-like macrophages characterization, and the use of the additional OVX+E2 experimental group which shows a reverse trend upon estrogen supplementation compared to the OVX group (n=3-4). Shown in (c) are the macrophages flow cytometry analysis results in FC1199 pancreatic liver metastasis model at Day 12 post-tumor injection (n=4).
