# Supplementary material for: Estrogen Receptor Blockade Potentiates Immunotherapy for Liver Metastases by Altering the Liver Immunosuppressive Microenvironment
Source: Cancer Res Commun. 2024 Aug 8;4(8):1963–77. doi: 10.1158/2767-9764.CRC-24-0196 (PMC11306998; doi:10.1158/2767-9764.CRC-24-0196)
Supplement: Table S1 — Immunofluorescence staining antibodies. [file crc-24-0196_table_s1_suppst1.pptx]

## Slide 1
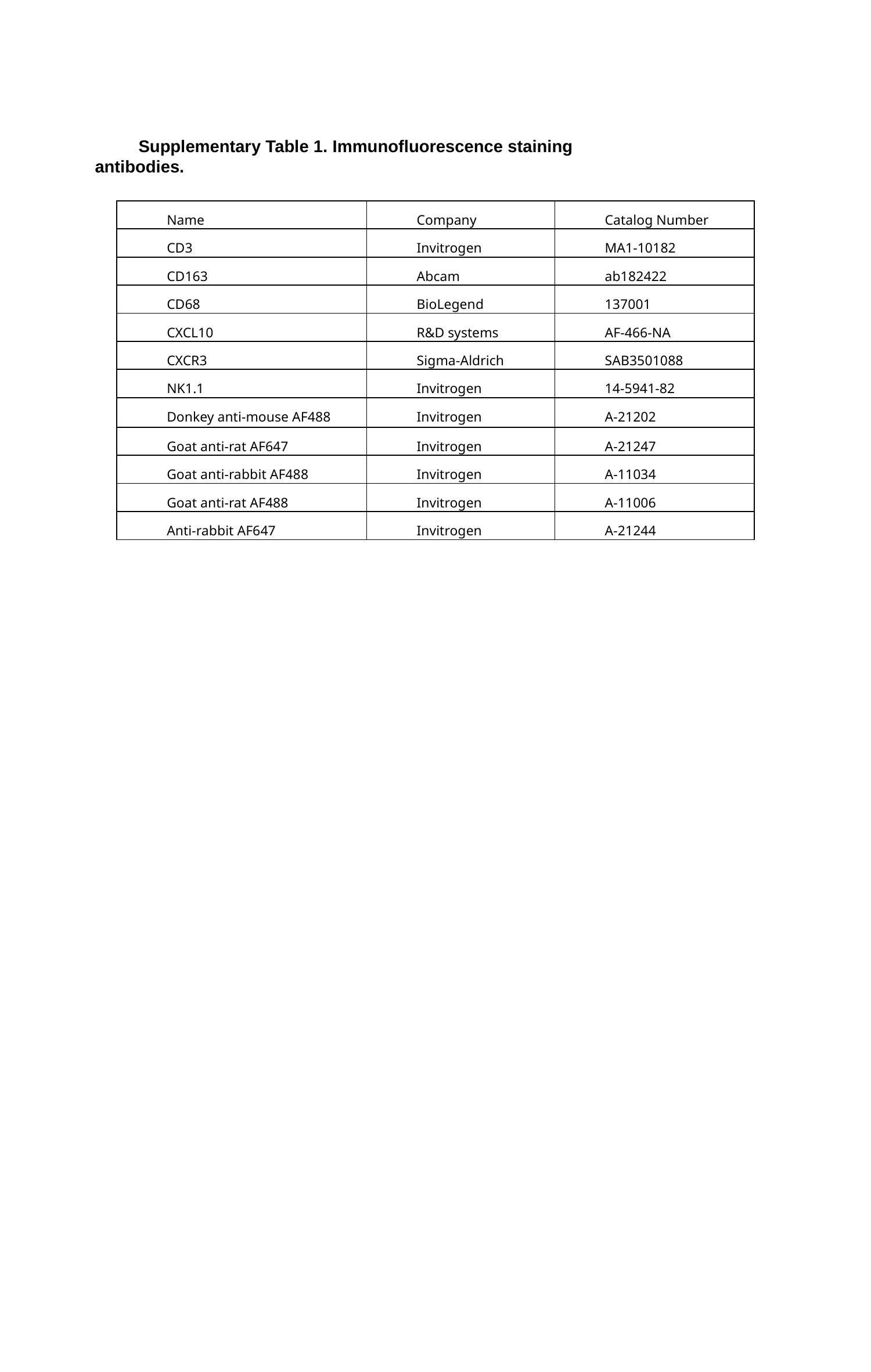

Supplementary Table 1. Immunofluorescence staining antibodies.
| Name | Company | Catalog Number |
| --- | --- | --- |
| CD3 | Invitrogen | MA1-10182 |
| CD163 | Abcam | ab182422 |
| CD68 | BioLegend | 137001 |
| CXCL10 | R&D systems | AF-466-NA |
| CXCR3 | Sigma-Aldrich | SAB3501088 |
| NK1.1 | Invitrogen | 14-5941-82 |
| Donkey anti-mouse AF488 | Invitrogen | A-21202 |
| Goat anti-rat AF647 | Invitrogen | A-21247 |
| Goat anti-rabbit AF488 | Invitrogen | A-11034 |
| Goat anti-rat AF488 | Invitrogen | A-11006 |
| Anti-rabbit AF647 | Invitrogen | A-21244 |
