# Supplementary material for: Estrogen Receptor Blockade Potentiates Immunotherapy for Liver Metastases by Altering the Liver Immunosuppressive Microenvironment
Source: Cancer Res Commun. 2024 Aug 8;4(8):1963–77. doi: 10.1158/2767-9764.CRC-24-0196 (PMC11306998; doi:10.1158/2767-9764.CRC-24-0196)
Supplement: Table S2 — Flow cytometry antibodies. [file crc-24-0196_table_s2_suppst2.pptx]

## Slide 1
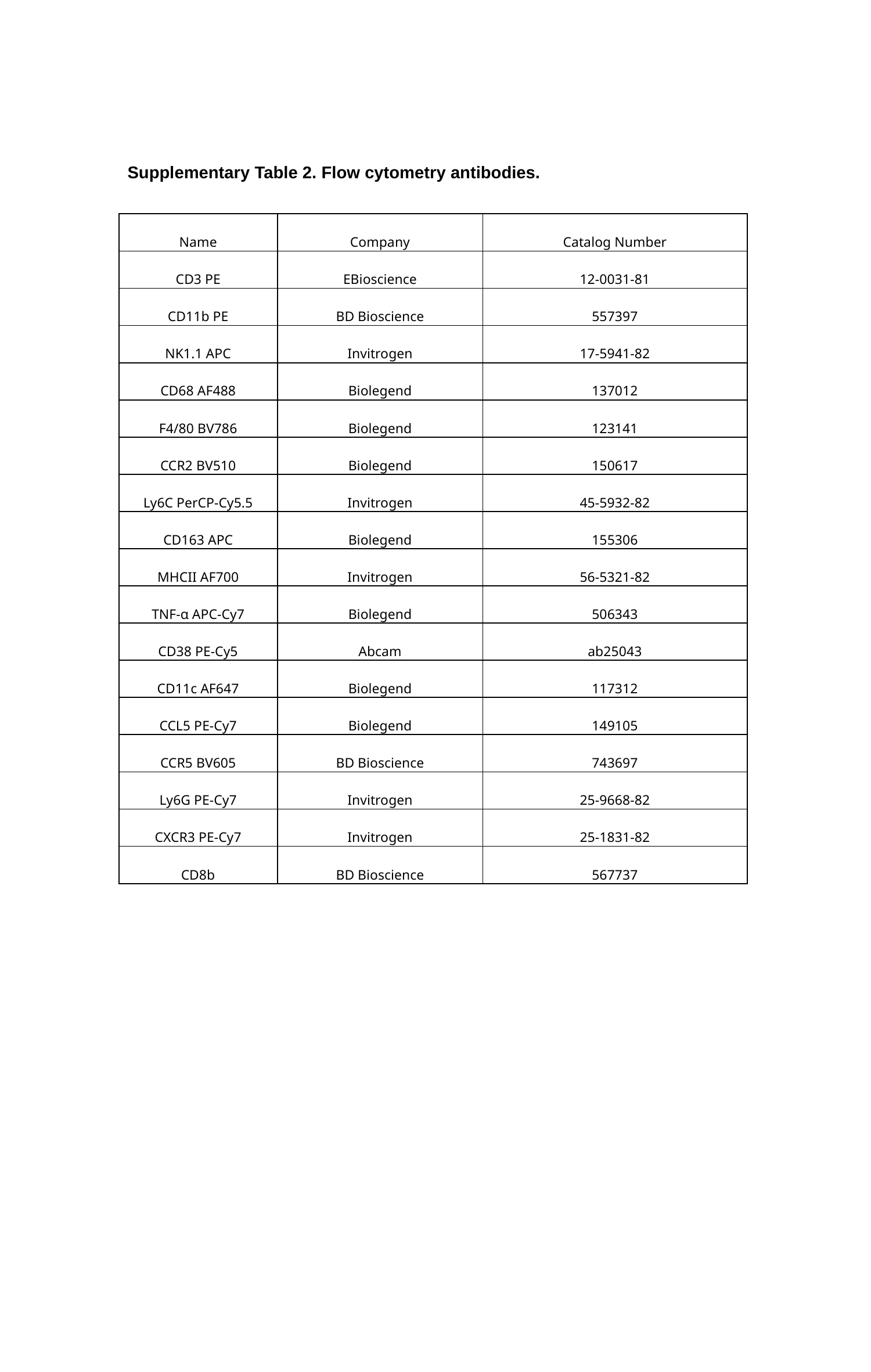

Supplementary Table 2. Flow cytometry antibodies.
| Name | Company | Catalog Number |
| --- | --- | --- |
| CD3 PE | EBioscience | 12-0031-81 |
| CD11b PE | BD Bioscience | 557397 |
| NK1.1 APC | Invitrogen | 17-5941-82 |
| CD68 AF488 | Biolegend | 137012 |
| F4/80 BV786 | Biolegend | 123141 |
| CCR2 BV510 | Biolegend | 150617 |
| Ly6C PerCP-Cy5.5 | Invitrogen | 45-5932-82 |
| CD163 APC | Biolegend | 155306 |
| MHCII AF700 | Invitrogen | 56-5321-82 |
| TNF-α APC-Cy7 | Biolegend | 506343 |
| CD38 PE-Cy5 | Abcam | ab25043 |
| CD11c AF647 | Biolegend | 117312 |
| CCL5 PE-Cy7 | Biolegend | 149105 |
| CCR5 BV605 | BD Bioscience | 743697 |
| Ly6G PE-Cy7 | Invitrogen | 25-9668-82 |
| CXCR3 PE-Cy7 | Invitrogen | 25-1831-82 |
| CD8b | BD Bioscience | 567737 |
