# Supplementary material for: Estrogen Receptor Blockade Potentiates Immunotherapy for Liver Metastases by Altering the Liver Immunosuppressive Microenvironment
Source: Cancer Res Commun. 2024 Aug 8;4(8):1963–77. doi: 10.1158/2767-9764.CRC-24-0196 (PMC11306998; doi:10.1158/2767-9764.CRC-24-0196)
Supplement: Table S3 — qPCR primer sequences. [file crc-24-0196_table_s3_suppst3.pptx]

## Slide 1
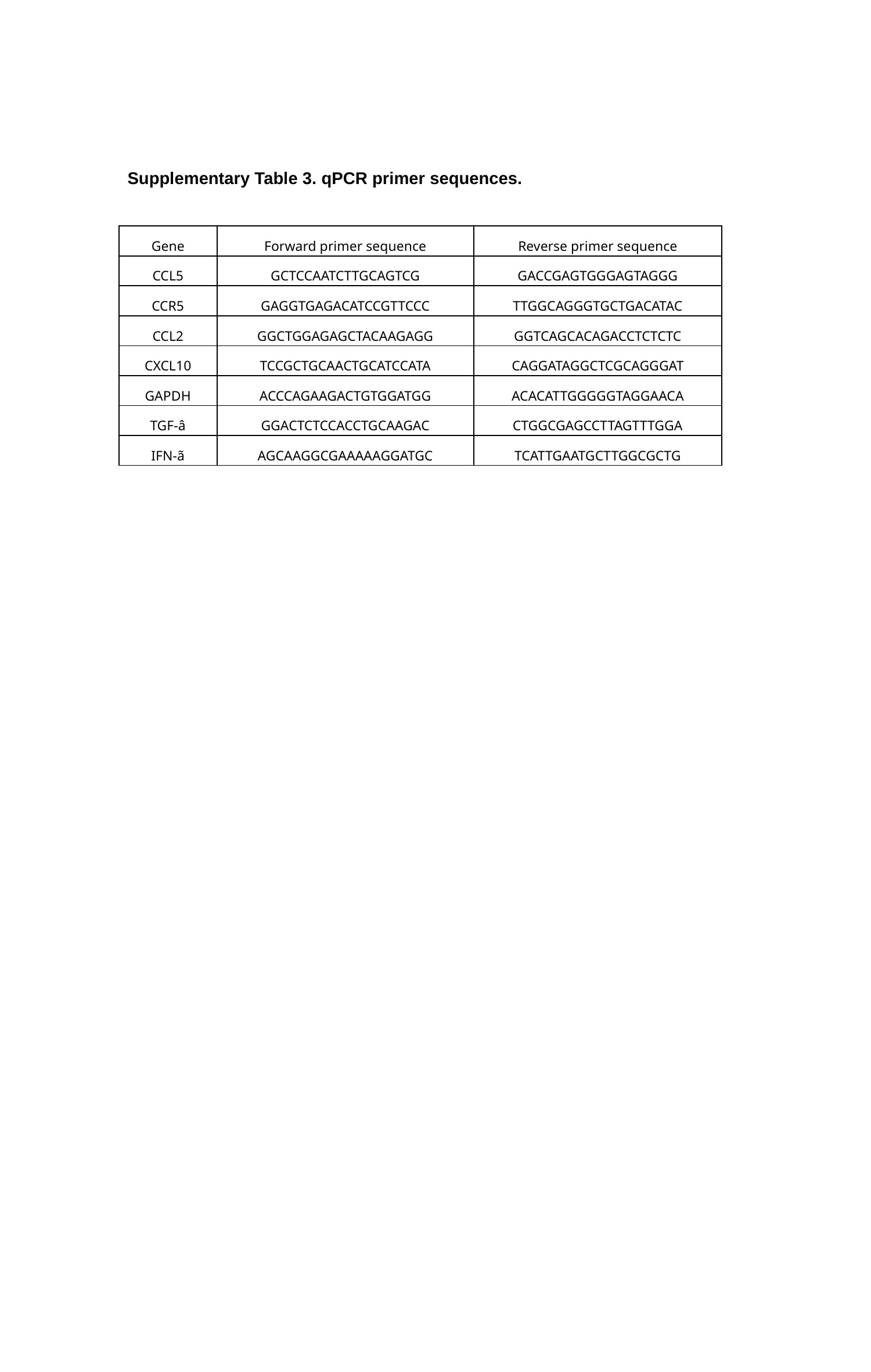

Supplementary Table 3. qPCR primer sequences.
| Gene | Forward primer sequence | Reverse primer sequence |
| --- | --- | --- |
| CCL5 | GCTCCAATCTTGCAGTCG | GACCGAGTGGGAGTAGGG |
| CCR5 | GAGGTGAGACATCCGTTCCC | TTGGCAGGGTGCTGACATAC |
| CCL2 | GGCTGGAGAGCTACAAGAGG | GGTCAGCACAGACCTCTCTC |
| CXCL10 | TCCGCTGCAACTGCATCCATA | CAGGATAGGCTCGCAGGGAT |
| GAPDH | ACCCAGAAGACTGTGGATGG | ACACATTGGGGGTAGGAACA |
| TGF-â | GGACTCTCCACCTGCAAGAC | CTGGCGAGCCTTAGTTTGGA |
| IFN-ã | AGCAAGGCGAAAAAGGATGC | TCATTGAATGCTTGGCGCTG |
